# Stiffness-induced cancer-associated fibroblasts are responsible for immunosuppression in a platelet-derived growth factor ligand-dependent manner

**DOI:** 10.1093/pnasnexus/pgad405

**Published:** 2023-12-18

**Authors:** Pia Gamradt, Kevin Thierry, Melissa Masmoudi, Zhichong Wu, Hector Hernandez-Vargas, Sophie Bachy, Tiffanie Antonio, Berkan Savas, Zainab Hussain, Richard Tomasini, Pascale Milani, Philippe Bertolino, Ana Hennino

**Affiliations:** Tumor Escape, Resistance and Immunity, Cancer Research Center of Lyon, UMR INSERM 1052, CNRS 5286, Lyon F-69373, France; Université Lyon 1, Lyon F-69000, France; Centre Léon Bérard, Lyon F-69008, France; Tumor Escape, Resistance and Immunity, Cancer Research Center of Lyon, UMR INSERM 1052, CNRS 5286, Lyon F-69373, France; Université Lyon 1, Lyon F-69000, France; Centre Léon Bérard, Lyon F-69008, France; Tumor Escape, Resistance and Immunity, Cancer Research Center of Lyon, UMR INSERM 1052, CNRS 5286, Lyon F-69373, France; Université Lyon 1, Lyon F-69000, France; Centre Léon Bérard, Lyon F-69008, France; StromaCare, Lyon F-69008, France; Tumor Escape, Resistance and Immunity, Cancer Research Center of Lyon, UMR INSERM 1052, CNRS 5286, Lyon F-69373, France; Université Lyon 1, Lyon F-69000, France; Centre Léon Bérard, Lyon F-69008, France; Department of General Surgery, Pancreatic Disease Center, Ruijin Hospital, Shanghai Jiao Tong University School of Medicine, Shanghai 200025, China; Tumor Escape, Resistance and Immunity, Cancer Research Center of Lyon, UMR INSERM 1052, CNRS 5286, Lyon F-69373, France; Université Lyon 1, Lyon F-69000, France; Centre Léon Bérard, Lyon F-69008, France; Tumor Escape, Resistance and Immunity, Cancer Research Center of Lyon, UMR INSERM 1052, CNRS 5286, Lyon F-69373, France; Université Lyon 1, Lyon F-69000, France; Centre Léon Bérard, Lyon F-69008, France; StromaCare, Lyon F-69008, France; Tumor Escape, Resistance and Immunity, Cancer Research Center of Lyon, UMR INSERM 1052, CNRS 5286, Lyon F-69373, France; Université Lyon 1, Lyon F-69000, France; Centre Léon Bérard, Lyon F-69008, France; Tumor Escape, Resistance and Immunity, Cancer Research Center of Lyon, UMR INSERM 1052, CNRS 5286, Lyon F-69373, France; Université Lyon 1, Lyon F-69000, France; Centre Léon Bérard, Lyon F-69008, France; INSERM 1068, CRCM, Marseille F-30059, France; INSERM 1068, CRCM, Marseille F-30059, France; Ecole Normale Supérieure de Lyon, Lyon F-69008, France; Tumor Escape, Resistance and Immunity, Cancer Research Center of Lyon, UMR INSERM 1052, CNRS 5286, Lyon F-69373, France; Université Lyon 1, Lyon F-69000, France; Centre Léon Bérard, Lyon F-69008, France; Tumor Escape, Resistance and Immunity, Cancer Research Center of Lyon, UMR INSERM 1052, CNRS 5286, Lyon F-69373, France; Université Lyon 1, Lyon F-69000, France; Centre Léon Bérard, Lyon F-69008, France; StromaCare, Lyon F-69008, France

**Keywords:** pancreatic adenocarcinoma (PDAC), cancer-associated fibroblasts (CAFs), tissue remodeling, PDGFR/PDGF signaling

## Abstract

Pancreatic ductal adenocarcinoma (PDAC) is associated with a vast stromal reaction that arises mainly from cancer-associated fibroblasts (CAFs) and promotes both immune escape and tumor growth. Here, we used a mouse model with deletion of the activin A receptor ALK4 in the context of the *Kras*^G12D^ mutation, which strongly drives collagen deposition that leads to tissue stiffness. By ligand–receptor analysis of single-cell RNA-sequencing data, we identified that, in stiff conditions, neoplastic ductal cells instructed CAFs through sustained platelet-derived growth factor (PDGF) signaling. Tumor-associated tissue rigidity resulted in the emergence of stiffness-induced CAFs (siCAFs) in vitro and in vivo. Similar results were confirmed in human data. siCAFs were able to strongly inhibit CD8^+^ T-cell responses in vitro and in vivo, promoting local immunosuppression. More importantly, targeting PDGF signaling led to diminished siCAF and reduced tumor growth. Our data show for the first time that early paracrine signaling leads to profound changes in tissue mechanics, impacting immune responses and tumor progression. Our study highlights that PDGF ligand neutralization can normalize the tissue architecture independent of the genetic background, indicating that finely tuned stromal therapy may open new therapeutic avenues in pancreatic cancer.

**Significance Statement**

Here, we report the identification of a cell population of cancer-associated fibroblast (CAF)-stiffness-induced CAFs (siCAFs) that is instructed by tumor cell through sustained platelet-derived growth factor (PDGF) signaling and that is able to strongly inhibit CD8^+^ T-cell response in vitro and in vivo. Our study provides support for the translational potential of using a PDGF ligand trap strategy in pancreatic cancer therapy.

## Introduction

Pancreatic ductal adenocarcinoma (PDAC) is currently the fourth leading cause of cancer-related death in the industrialized world and is predicted to become the second leading cause of cancer-related death by 2030 (1). PDAC develops through the preceding formation of acinar-to-duct metaplasia (ADM) and pancreatic intraepithelial neoplasia (PanIN), which are primarily driven by oncogenic Kras activation (2). In addition, PDAC is associated with an abundant stromal reaction that usually surrounds islands of cancer cells and accounts for 50–80% of the tumor volume (3, 4).

The pancreatic tumor stroma consists of a variety of cellular and noncellular components. A broad range of extracellular matrix (ECM) proteins, such as collagens, fibrous and nonfibrous glycoproteins, and proteoglycans contribute to the structural formation of the noncellular stromal compartment. In addition, the ECM also contains nonstructural components, such as growth factors and matricellular proteins (4–6). The cellular compartment of the stroma includes immune cells, such as lymphocytes, macrophages, mast cells, and myeloid-derived suppressor cells (MDSCs), along with vascular and neural elements (endothelial cells and neurons, respectively) (7–9).

Accumulating evidence indicates the presence of close and complex paracrine interactions mediating bidirectional crosstalk between tumor cells and the cellular and noncellular stroma that facilitates cancer progression (5). While the stroma might provide a barrier limiting the dissemination and metastasis of pancreatic cancer cells, it also stimulates aggressive behaviors in pancreatic cancer cells and helps these cells escape host immune surveillance (10, 11). Mechanical tissue stiffness is associated with poor survival in PDAC patients (12–14). It is now a well-established fact that activated pancreatic stellate cells (PSCs) are primarily responsible for the development of the stroma (15). PSCs represent ∼4% of all pancreatic cells in the steady state. Upon inflammation, PSCs are activated and converted into cancer-associated fibroblasts (CAFs), which are the main source of ECM proteins and growth factors (16). Several mouse studies have shown that CAF depletion abolishes immune suppression (17). Surprisingly, contrary to the initial preclinical results (18), several publications have shown that the stromal response mediated by hedgehog signaling inhibits tumor progression and that its ablation would be harmful in PDAC. However, it has been shown that high stromal activity, as represented by α-smooth muscle actin (α-SMA) expression, is associated with a poor prognosis in patients with pancreatic cancer (3). All these results show that tumor–stroma interactions are complex. Indeed, several populations of CAFs with different functions related to antitumor immune responses have been described in both breast cancer (19) and pancreatic cancer (20, 21), indicating that the modulation of stromal activity rather than overall depletion of the stroma would be a therapeutic approach of choice.

Members of the Transforming Growth Factor (TGF)-β superfamily, including TGF-β, activins, inhibins, bone morphogenic proteins, growth and differentiation factors and nodal, have growth-stimulatory or growth-inhibitory effects in different types of tumors (22). Inactivating mutations in ALK4, the receptor of activin A, have been identified in pancreatic cell lines derived from patients (23, 24). These mutations are associated with increased tumor aggressiveness and a poor survival prognosis (23). Recently, our group discovered that activin A secreted by neoplastic cells acts as a protective senescence-associated secretory phenotype protein that limits tumor progression even during the early stage of ADM by preventing massive ECM deposition (25). Here, we aimed to investigate the role of early tissue mechanical alterations in driving CAF differentiation and the consequent impact on the immune response.

## Methods

### Mouse models

All animal protocols were reviewed and approved in accordance with the guidelines provided by the CRCL Animal Care Committee (CECCAPP_CLB_2019_002). The generation of Acvr1b^flox/flox^ mutant mice has been previously described (26). Acvr1b^flox/flox^; LSL-Kras^G12D/+^; Ptf1a-Cre mice (termed 4KC mice) were generated by crossing Acvr1b^flox/flox^ mice with previously established LSL-Kras^G12D/+^; Ptf1a-Cre mice (termed KC mice) (27).

### Atomic force microscopy

Detailed information about atomic force microscopy (AFM) analysis is given in supplementary methods.

### Flow cytometric analysis

Detailed information about the flow cytometric analysis are given in supplementary methods.

### Single-cell RNA sequencing

Detailed information about single-cell RNA sequencing (scRNAseq) are given in supplementary methods.

### LEGENDplex custom array

The experiment has been carried out according to the provider's protocol.

### Statistical analysis

GraphPad Prism was used for the graphical representation of the data and statistical analysis. *P*-values were calculated using Student's test. For multiple comparisons, a one-way analysis of variance with Tukey's post hoc test was used. Significance was indicated as follows: *P < 0.05, **P < 0.01, ***P < 0.001, and ****P < 0.0001.

## Results

### ALK4 signaling disruption in neoplastic cells leads to early increased collagen deposition and tissue rigidity

**Fig. 1.**
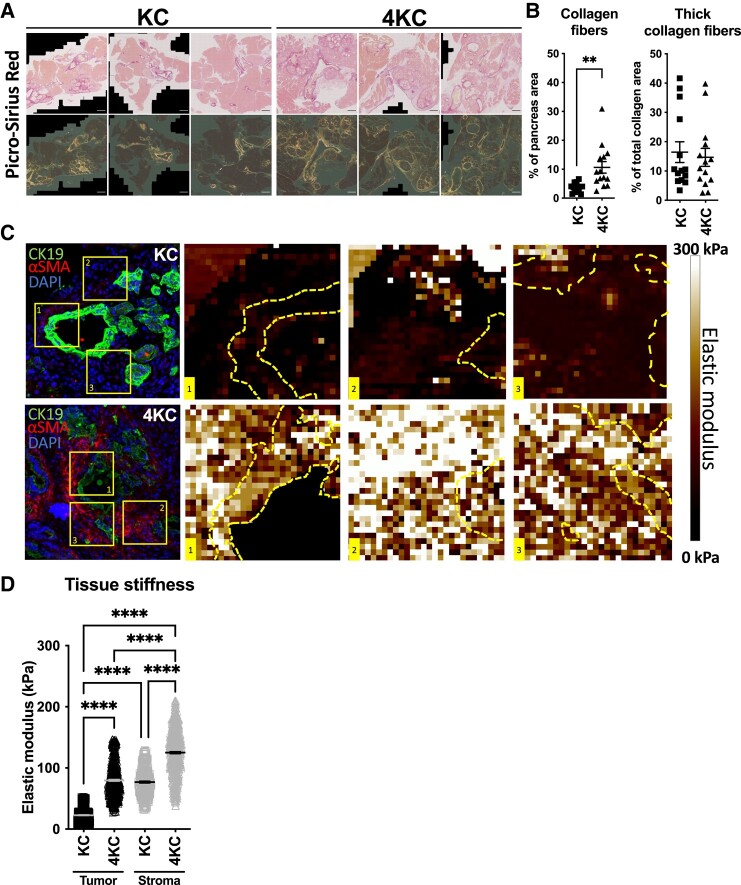
ALK4 signaling disruption in tumor cells alters the tissue mechanics of the pancreatic TME. A) Representative photographs of pancreata from 6-week-old KC (left panel) and 4KC mice (right panel) stained with Picrosirius red. Total collagen (top: transmitted light) and thick collagen fibers (bottom: polarized light) are shown. B) Quantification of the total collagen (left) and thick collagen fiber (right) content in the pancreata of KC and 4KC mice. Cumulative data from three individual experiments with four to five mice per group are shown. C) IF staining of pancreata from 6-week-old KC and 4KC mice for CK19 (green), α-SMA (red; KC mice), and 4′,6-DIamidino-2-PhenylIndole (DAPI) (blue). Stiffness topography measured by AFM of selected regions (1, 2, or 3 as indicated on the IF staining images) in pancreata from KC and 4KC mice. D) Quantification of the elastic modulus (kPa) measured by AFM in tumor or stromal regions of KC and 4KC pancreata. Cumulative data from three independent mice per group are shown. One hundred force curves per zone of interest were measured. B and D) The mean values ± SEMs are displayed. **P < 0.01; ****P < 0.0001.

Given that ablation of protective activin A signaling promotes the formation of ADM lesions (25), we sought to further evaluate the effect of ALK4 signaling disruption in neoplastic cells on the structural and mechanical qualities of the pancreatic tumor microenvironment (TME). Although oncogenic KRAS^G12D^ expression occurs during the prenatal state in both KC mice and 4KC mice (28), ADM lesions develop only at or shortly after the time of weaning. While no difference in pancreas weight was observed at 3 weeks of age, at 6 weeks of age, the pancreata of 4KC mice were already significantly enlarged than those of KC mice (Fig. S1A and B). Histological analysis revealed significantly expanded areas of pancreatic lesions in 4KC mice compared with KC mice at 6 weeks of age, and while this expansion was mainly mediated by stroma formation, ADM formation was also accelerated in 4KC mice at this age (Fig. S1C and D). In both KC and 4KC pancreata, collagen deposition was observed in the stromal compartment of lesions, as determined by histological analysis of Sirius red staining (Fig. 1A), and as expected, the total collagen amount was significantly higher in the 4KC pancreata (Fig. 1B), which was accompanied by palpable tissue induration. Interestingly, although more collagen was detected in 4KC pancreata than in KC pancreata, evaluation of Sirius red staining under polarized light revealed no change in the thickness of the collagen fibers for either genotype (Fig. 1B).

To compare the rigidity within distinct tissue compartments between KC and 4KC pancreata, we performed AFM analysis in combination with immunofluorescence (IF) microscopy. Stromal and ADM regions were identified by the expression of α-SMA and CK19, respectively, and the elastic modulus was measured in three different regions per sample (Fig. 1C and D). The data revealed increased tissue rigidity in the stroma of 4KC mice compared with that of age-matched KC mice (Fig. 1D), which could be explained by increased deposition of collagen fibers that most likely form more interfiber connections, which create tissue stiffness (Fig. 1A and B). Of note, we also detected an increase in tissue stiffness in the neoplastic compartment of 4KC mice compared with that of KC mice (Fig. 1D). Taken together, the results indicate that disruption of ALK4 signaling in neoplastic cells not only overcomes protective antitumorigenic mechanisms as previously shown (25) but also produces a strong paracrine effect at the early stage of ADM, resulting in excessive ECM secretion within the TME.

### Sustained PDGF signaling is increased in stiff tissue conditions

**Fig. 2.**
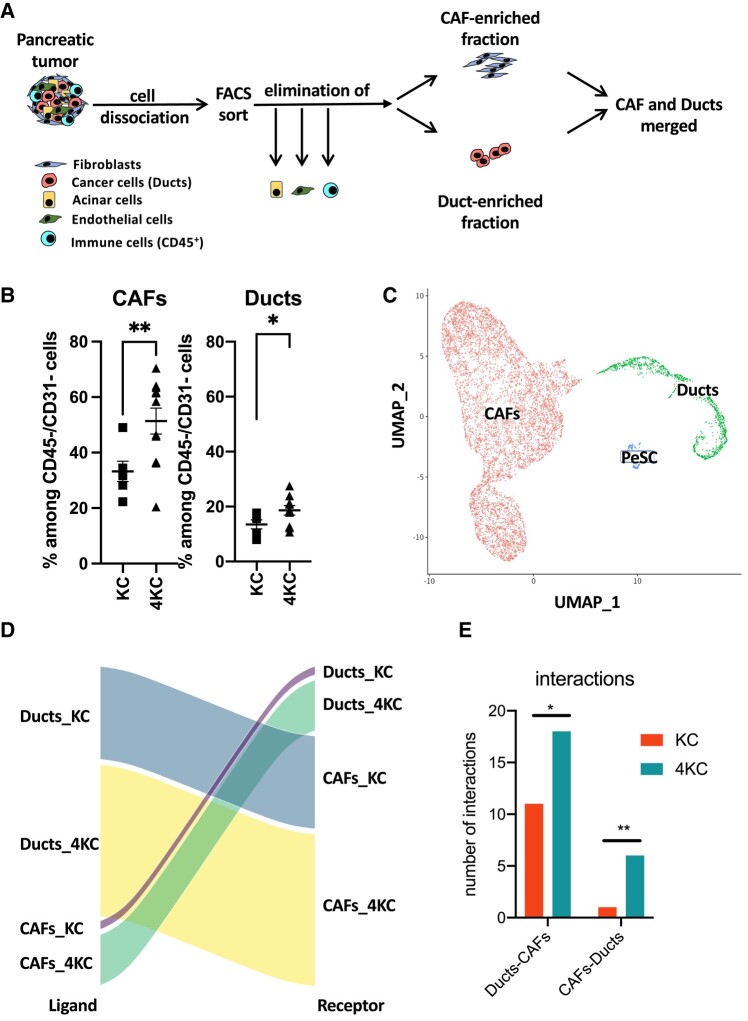
Identification of increased interactions between components in stiff conditions. (A) Graphical scheme representing the workflow for CAF and duct isolation from the pancreata of 6-week-old KC and 4KC mice by FACS sorting. Both sorted fractions were further subjected to single-cell capture, barcoding, and reverse transcription using the 10× genomics platform. B) Frequencies of CAFs (lectin PNA-EpCAM–) and ductal cells (Lectin PNA-EpCAM+) among viable CD45-CD31 cells detected by FACS analysis. The mean values ± SEMs are displayed. *P < 0.05, **P < 0.01. C) Uniform Manifold Approximation and Projection (UMAP) plot of merged sorted fractions illustrating the CAF, duct, and PeSC fractions. D) Ligand:Receptor network maps were obtained from scRNAseq data using “SingleCellSignalR.” Alluvial plot representing the interactions between ducts and CAFs separately for the two conditions (i.e. KC and 4KC). Paracrine signals flow from ligands (left column) to receptors (right column), with the width of the connecting streams indicating the number of interactions. E) Number of interactions (ducts-CAFs and CAFs-ducts) in 4K and 4KC conditions. *P < 0.05; **P < 0.01, two-sided test of proportions.

Recently, the complex heterogeneity of CAFs has been revealed by other studies, indicating the existence of CAF subpopulations equipped with pro- and antitumorigenic qualities (19–21, 29). Although CAFs have been determined to be the main ECM producers, to our knowledge, there is no data available linking stromal tissue stiffness to the phenotypic and functional properties of CAFs after their initial instruction/activation by neoplastic cells. Thus, we took advantage of the KC and 4KC mouse models representing opposing ends of the tissue stiffness scale and performed scRNAseq analysis of pancreatic CAFs and neoplastic enriched cell fractions. Fluorescence activated cell sorting (FACS) was used to exclude hematopoietic and endothelial cells based on their expression of CD45 and CD31, respectively. Next, lectin peanut agglutinin (PNA)^+^ acinar cells were excluded (30) to enrich for CAFs (CD45^−^CD31^−^Lectin PNA-EpCAM^−^) or neoplastic ductal cells (CD45^−^CD31^−^Lectin PNA^−^EpCAM^+^; (Fig. 2A and S2A). Importantly, in 4KC mice, CAFs, and ductal cells were present at significantly higher frequencies among pancreatic CD45-CD31 cells (Fig. 2B), but equal numbers of single cells from each sample (KC or 4KC) and each fraction (CAFs or ducts) were captured and sequenced using a droplet-based approach.

In the CAFs-enriched fraction, nine main clusters (CAFs 0–8) were identified in both KC mice and 4KC mice and the cluster proportions are indicated (Fig. S2B). We identified cluster 7 as being pericyte stem cells (PeSCs) that we recently identified in neoplasia (31). Previously described myofibroblast CAFs (myCAFs), inflammatory CAFs (iCAFs), and antigen-presenting CAFs (apCAFs) (21) signatures were identified as clusters 6, 3, and 8 in both conditions (Fig. S2C–E). All of the previously identified populations of CAFs were significantly increased in proportions in the 4KC condition compared with KC (Fig. S2B).

In order to identify any particular interactions between the CAF and duct cell population associated with increased tissue stiffness, we merged the two fractions and obtained three major clusters: CAFs, ducts (tumor), and in addition, a small fraction of PeSCs (Fig. 2C). To determine how these three populations interact, we performed ligand-receptor-based SingleCellSignalR algorithms (32) analysis among the CAFs, ducts, and PeSCs in the KC and 4KC conditions. The algorithm was used in “paracrine” mode with a large receptor:ligand database specific for mouse, as previously described (32). Our analysis revealed that there were increased number of interactions among the 3 clusters in the KC soft condition than in the 4KC stiff condition (Fig. S2F) and the specific top 50 interactions are represented (Fig. S2H). To test the hypothesis that early instruction from duct cells to CAFs contributes to the establishment of stiff phenotype, we analyzed the interactions between ducts and CAFs in both soft (KC) and stiff conditions (4KC). As shown in Fig. 2D and E, we determined an increased number of interactions either from ducts toward CAFs or from CAFs toward ducts in 4KC condition.

**Fig. 3.**
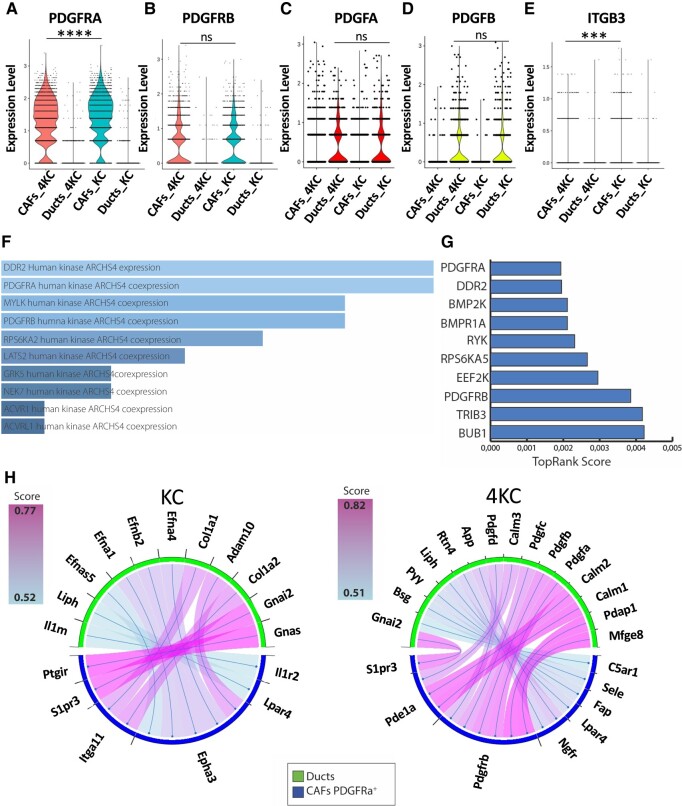
Identification of PDGF signaling signature associated with tissue stiffness. A–E) Violin plots displaying the gene expression of PDGFRA, PDGFRB, PDGFA, PDGFB, and ITGB3 by CAFs and ducts obtained from 6-week-old KC and 4KC mice determined by scRNAseq (C–G). ***P < 0.001; ****P < 0.0001; ns, not significant. F) Top signaling term enrichment of the 29-gene siCAF signature according to the massive mining of publicly available RNA-Seq data from human and mouse ARCHS4 database (https://maayanlab.cloud/archs4/). G) Top signaling term enrichment of the 29-gene siCAF signature according to the kinase enrichment analysis version 3 (KEA3) database. (https://maayanlab.cloud/kea3/). H) Receptor–ligand interactions (as assessed with SingleCellSignalR) from ductal to PDGFRA + CAFs in KC (left) and 4KC (right) conditions.

We analyzed in detail the most upregulated interactions in both KC and 4KC conditions (Fig. S2H). We identified platelet-derived growth factor (PDGF)-dependent interactions from ducts to CAFs to be prevalent in 4KC while absent in KC. As PDGFs are considered fibrogenic growth factors, we next sought to determine the expression of PDGFRAA, PDGFRB, PDGFA, and PDGFB in merged scRNAseq data. While the two major receptors, PDGFRA and PDGFRB, were exclusively expressed in the CAF compartment, the ligands PDGFA and PDGFB were exclusively expressed in the duct compartment (Fig. 3A–D). In addition, we analyzed the expression of the integrin (ITG) β3/CD61, which has been shown to be key in CAF-mediated tumor cell invasion via the assembly of the ECM protein fibronectin (33) and thereby might promote tissue stiffness. Moreover, CD61 is known to interact with the ECM protein βig-h3/TGF-βi (34), which has been described as a key ECM protein in the pancreatic TME hampering conventional (35, 36) and unconventional T-cell responses (37). We observed that CD61 was also expressed in the CAF compartment (Fig. 3E).

As shown in Fig. 3A, most CAFs expressed PDGFRA in both KC and 4KC conditions (82 and 74% of CAFs, respectively). Therefore, we performed differential expression analysis in 4KC vs. KC, using only PDGFRA^+^ CAFs. This analysis resulted in 72 differentially expressed genes (DEGs), 29 of them upregulated, and 43 downregulated in 4KC CAFs. DEGs were tested for enrichment in Gene Ontology, Kyoto Encyclopedia of Genes and Genomes (KEGG), and REACTOME pathway terms showing significant differences in ECM organization, assembly of collagen fibrils, and collagen formation (Fig. S3A–C) in the 4KC stiff relative to KC soft condition. Furthermore, most of the ECM pathways from several repositories were enriched in the DEGs of the PDGFRα^+^ vs. PDGFRα^−^, suggesting these pathways exhibit dependence on PDGFRA expression (Fig. S3D–F). Based on the 29 4KC upregulated genes (Table S1) we generated a stiffness CAF signature. To specifically test for potential signaling activity, we interrogated the 29-gene CAF signature for kinase activity enrichment using 2 different kinase databases. In both cases, PDGFRA was among the top kinase activities significantly enriched in the CAF signature (Fig. 3F and G). Furthermore, ligand–receptor interaction analysis was also performed separately for PDGFRA^+^ positive CAFs. More PDGF-related interactions from ducts to CAFs were detected in the 4KC condition, relative to the KC condition (Fig. 3H). We further looked for the distribution of stiffness-induced CAFs (siCAFs) signature across the nine CAF clusters. We applied the 29-gene siCAF signature using the AUCell method to calculate a score for every single cell (38). As shown in Fig. S3G, all clusters displayed at least one fraction of cells with relatively high score with the exception of cluster 7, previously identified as PeSC (31). Altogether, these results indicate that PDGF–PDGFR interactions play a key role in the early establishment of tissue stiffness independent of CAF origin and subtype.

### Increased tissue stiffness is associated with the loss of PDGFRα surface expression on CAFs

**Fig. 4.**
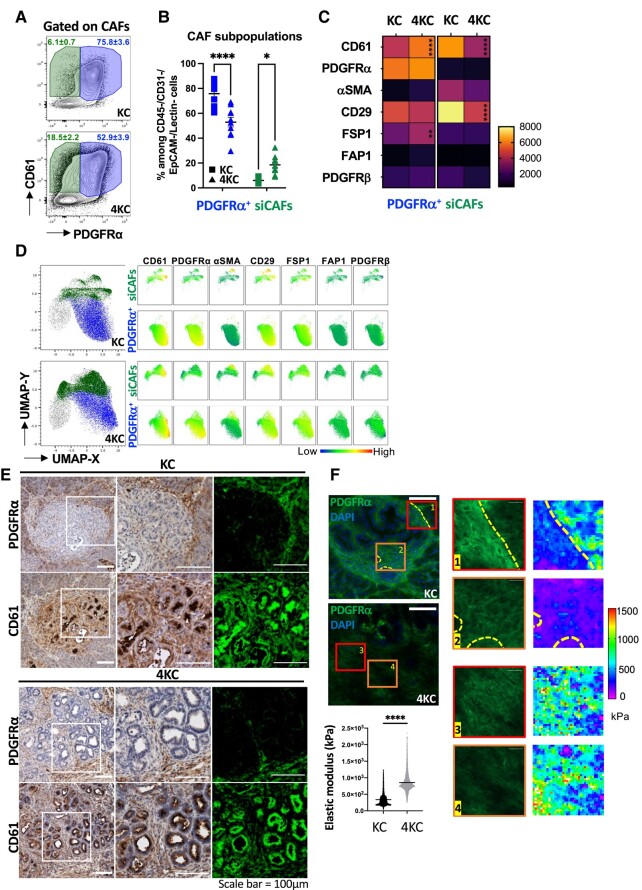
Increased tissue stiffness favors the accumulation of siCAFs. A) Representative FACS dot plots showing the surface expression of PDGFRα and CD61 on PDGFRα^+^ CAFs and siCAFs (PDGFRα^−^) in pancreata from 6-week-old KC (top) and 4KC (bottom) mice. Cells were gated on viable CD45^−^CD31^−^Lectin PNA^−^EpCAM^−^ CAFs. B) Frequencies of PDGFRα^+^ CAFs and siCAFs among CD45^−^CD31^−^Lectin PNA^−^EpCAM^−^ CAFs in pancreata from 6-week-old KC (squares) and 4KC mice (triangles). C) Heatmap of the MFIs of CAF markers on PDGFRα^+^ CAFs and siCAFs from KC and 4KC pancreata. D) Unsupervised UMAP analysis of CAFs from KC (top) and 4KC (bottom) pancreata, with PDGFRα^+^ CAFs and siCAFs highlighted in blue and green, respectively, and corresponding multigraph mapping of CAF markers for each cell population. E) Representative images of IHC staining for PDGFRα (top) and CD61 (bottom) in serial sections of pancreata from 6-week-old KC (left panel) and 4KC (right panel) mice. White squares indicate the magnified regions of each panel. The images on the right of each panel show pseudocolored positive cells for the indicated marker (green). F) Stiffness topography measured by AFM of selected regions (1–4 as indicated on the IF staining images) in pancreata from KC and 4KC mice. Quantification of the elastic modulus (kPa) measured by AFM in stromal regions of KC and 4KC pancreata. Scale bars 100 and 20 mm. A–F) Cumulative data from three individual experiments with three to four mice per group are shown. The mean values ± SEMs are displayed. *P < 0.05; **P < 0.01; ****P < 0.0001.

Based on these observations, we sought to further examine the TME of 4KC and KC mice by determining the phenotype of CAFs by multicolor flow cytometry. Therefore, we applied the same gating strategy as that used in FACS sorting and focused on the CAF populations. FACS analysis of the two markers PDGFRα and CD61 identified a CAF population that was positive for both markers (PDGFRα^+^CD61^+^) and present in both KC mice and 4KC mice at frequencies of 75.8 and 52.9%, respectively (Fig. 4A and B). In addition, we detected a PDGFRα^−^CD61^+^ CAF population that was significantly increased in 4KC pancreata (18.5%) compared with KC pancreata (6.1%; Fig. 4A and B). Given the association of these 2 CAF populations with the opposing tissue stiffness explored in KC and 4KC mice, we termed them siCAFs and PDGFRα^+^ CAFs. The fact that we did not detect the siCAF population by scRNAseq analysis highlighted that the disruption of ALK4 signaling in 4KC mice might be a result of continuous signaling through the PDGF–PDGFR system at the protein level.

In addition to PDGFRα and CD61, we further evaluated the expression of the established CAF markers α-SMA, Fibroblast activation protein (FAP)1, FSP1/S100-A4, and PDGFRα, as well as CD29/ITGβ1 (19, 39). Interestingly, α-SMA and FSP1, which are both considered activated CAF markers (40, 41), were expressed on siCAFs and PDGFRα^+^ CAFs, respectively. While no differences in the expression of α-SMA were found between KC and 4KC siCAFs, FSP1 expression was significantly increased on PDGFRα^+^ CAFs from 4KC mice (Fig. 4C). Moreover, the expression of the integrins CD61 and CD29 was significantly higher on PDGFRα^+^ CAFs obtained from KC mice. In contrast, the mean fluorescence intensity (MFI) of CD61 was significantly higher on PDGFRα^+^ CAFs from 4KC mice (Fig. 4C). Unsupervised t-distributed stochastic neighbor embedding analysis of siCAFs and PDGFRα^+^ CAFs revealed an increased siCAF population in the high-stiffness conditions (Fig. 4D). Of note, we also detected a PDGFRα^−^CD61^−^ CAF population in both KC and 4KC pancreata, representing 16.8 and 26.7% of all CAFs, respectively (Fig. S4A). However, since this population did not express any of the other evaluated CAF markers (Fig. S4B), we excluded it from this study. Nevertheless, given the relatively high frequency, this population should be subject to future analysis.

Spatial analysis by immunohistochemistry (IHC) displayed an almost exclusive site of PDGFRα^+^ stromal cells at the outer edge of pancreatic lesions that was more pronounced in 4KC than in KC pancreata (Fig. 4E). We detected a diminished staining for PDGFRα protein in 4KC although the PDGFRα mRNA detected by RNAscope was similar in both conditions (Fig. S4C–E). Linking the spatial distribution of PDGFRα and CD61 expression with the phenotype determined by FACS analysis, we concluded that PDGFRα^+^ CAFs were located at the edge of lesions, whereas siCAFs were mainly found within 4KC lesion centers. To compare the rigidity within distinct PDGFRα^+^ and PDGFRα^−^ regions between KC and 4KC pancreata, we performed AFM analysis in combination with IF microscopy. We detected increased elastic modulus in PDGFRα^−^ stromal area in 4KC mice. In KC mice, PDGFRα^+^ area had a significant diminished elastic modulus compared with the PDGFRα^−^ area (Fig. 4F). These results demonstrate that siCAFs are localized in the stiff regions in mouse pancreata. Taken together, our data demonstrate that stiffness-promoting ductal cells lead to specific instruction of CAFs, characterized by a unique phenotypic signature based on two markers: PDGFRα and CD61.

### Loss of PDGFRα surface expression on siCAFs is a tumor cell-driven early event accompanied by PDGF ligand accumulation

**Fig. 5.**
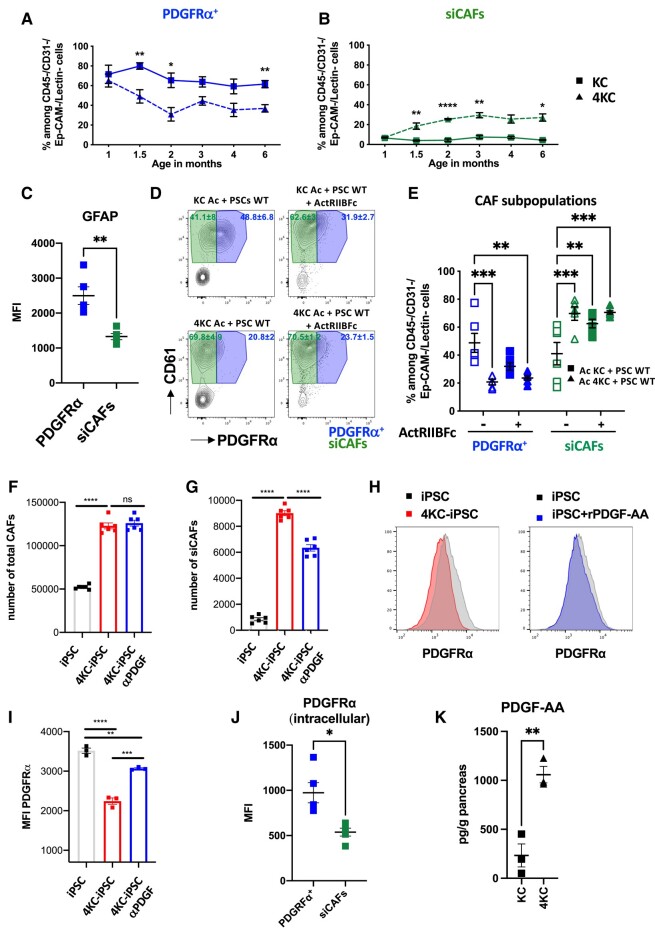
Neoplastic cells instruct the emergence of siCAFs at early tumor stages. A and B) Frequencies of (A) PDGFRα^+^ CAFs and (B) siCAFs among CD45^−^CD31^−^Lectin PNA^−^EpCAM^−^ CAFs determined by FACS analysis of pancreata from KC (squares) and 4KC mice (triangles) harvested at 1, 1.5, 3, 4, and 6 months of age. C) MFI of intracellular GFAP in PDGFRα^+^ CAFs and siCAFs determined by FACS analysis. D) Representative FACS dot plots showing the surface expression of PDGFRα and CD61 on PDGFRα^+^ CAFs and siCAFs generated by coculturing WT PSCs with acinar cells (Ac) from KC or 4KC mice in the absence or presence of the soluble activin A inhibitor ActRIIBFc. E) Frequencies of PDGFRα^+^ CAFs and siCAFs generated in the absence or presence of ActRIIBFc. F and G) siCAFs were generated by coculturing iPSCs with 4KC cell line in the absence or presence of αPDGF Ab for 7 days. F) Number of iPSC and total CAFs and (G) number of siCAFs determined by FACS analysis. H and I) siCAFs were generated by coculturing iPSCs with 4KC cell line or in the presence of rPDGF-AA for 7 days. H) Histograms of PDGFRα expression. I) MFI of extracellular PDGFRα determined by FACS analysis. J) MFI of intracellular PDGFRα in PDGFRα^+^ CAFs and siCAFs determined by FACS analysis. K) Tissue levels of PDGF-AA (pg/g) determined by ELISA analysis of pancreata from 6-week-old KC and 4KC mice. A and B) Results from five mice per group and time point are shown. C and F) Representative data from two individual experiments with five mice are shown. G) Representative data from two individual experiments with three mice and technical triplicates are shown. F–J) Representative data from two individual experiments and technical triplicates are shown. The mean values ± SEMs are displayed. *P < 0.05; **P < 0.01; ***P < 0.001; ****P < 0.0001.

To better understand the instruction of CAFs by ductal cells and the contribution of CAFs to altered tissue mechanics, we determined the kinetics of PDGFRα^+^ and siCAFs emergence in KC and 4KC mice by FACS performed at 1, 1.5, 2, 3, 4, and 6 months of age. In KC mice, the frequencies of PDGFRα^+^ CAFs and siCAFs stably represented ∼66.9 and 5.6% of all CAFs, respectively (Fig. 4A and B). In contrast, although the frequencies in 4KC mice were comparable with those in KC mice at 1 month of age, a significant decrease and increase in PDGFRα^+^ CAFs and siCAFs, respectively, could be observed as early as 1.5 months of age. Moreover, these changes in the CAF populations of 4KC mice stabilized until the end of the experiment at 6 months of age, with PDGFRα^+^ CAFs representing 36.9% and siCAFs representing 27.1% of all CAFs (Fig. 5A and B). Notably, the previously mentioned PDGFRα-CD61-CAF population remained unchanged in both KC and 4KC mice over the course of the experiment (Fig. S5A). These results indicate that early CAF instruction was maintained and that PDGFRα^+^ CAFs and siCAFs most likely represented two different CAF activation states associated with the opposing tissue stiffness phenotypes of KC and 4KC mice. In line with these findings, we observed that siCAFs had significantly lower expression of the qPSC marker glia fibrillary acidic protein (GFAP) than PDGFRα^+^ CAFs (Fig. 5C), further highlighting the different activation statuses of these two cell populations.

We next aimed to verify that the instruction of PDGFRα^+^ CAFs into siCAFs in 4KC mice is driven through altered signals mediated by neoplastic ductal cells lacking ALK4 signaling rather than by increased release and/or availability of the ALK4 ligand activin A, which is still produced in ALK4-knock out (KO) tumor cells. Therefore, we differentiated PSCs isolated from wild-type (WT) mice into CAFs in the presence of neoplastic cells from KC or 4KC pancreata and added ActRIIBFc, a soluble inhibitor of activin A. Previously, injection of ActRIIBFc into KC mice has been shown to result in a 4KC-like phenotype (25). Similar to our previous ex vivo data, even in the absence of ActRIIBFc, PSCs differentiated into more siCAFs when cocultured with 4KC acinar cells rather than KC acinar cells, which produced significantly more PDGFRα^+^ CAFs (Fig. 5D, left panel, and E). However, when ActRIIBFc was added to the coculture containing KC acinar cells, the frequency of siCAFs was similar to that observed in the coculture containing 4KC acinar cells. The addition of ActRIIBFc to the coculture containing 4KC acinar cells did not affect the expression of CD61 or PDGFRα on CAFs (Fig. 5D, right panel, and E).

We next aimed to investigate the reason for the downregulation of PDGFRa surface expression in 4KC CAFs as an indicator of CAF activation. PDGFRs are internalized after binding to their ligands. First, we verified that PDGFRα was phosphorylated in PSCs after ligand binding in vitro. As neoplastic primary cells from 4KC mice as well as primary PSC from WT are difficult to generate in high numbers, we used alternatively immortalized PSC (iPSC) previously described (20) as well as 4KC-green fluorescent protein (GFP) primary cell line generated as previously described (31). iPSC displayed increased phosphorylation upon 4KC culture media or recombinant PDGRF-AA binding (Fig. S5D and E). Next, we performed in vitro generation of siCAF for 7 days in presence or absence of a neutralizing polyclonal anti-PDGF antibody. As shown in Fig. S5F and G the use of anti-PDGF neutralizing Ab was able to significantly reduce the phosphorylation of PDGFRα. Moreover, the number of siCAF diminished in the presence of anti-PDGF neutralizing Ab (Fig. 5F and G). Adding recombinant PDGRF-AA induced PDGRFa surface expression downregulation on the surface of CAFs, although to a lesser extent than in the presence of a 4KC-GFP cell line (Fig. 5H and I). Ultimately, most internalized PDGFRs are subjected to lysosomal or proteasomal degradation (42). FACS analysis revealed that siCAFs had lower intracellular PDGFRa expression than PDGFRα^+^ CAFs (Fig. 5J) and the use of MG-132 proteasome inhibitor increased the accumulation of PDGFRα (Fig. S5H and I). We speculated that the increased availability of PDGF ligands in 4KC mice results in receptor internalization, which in turn promotes cell proliferation and ECM deposition (43–45). Although PDGF ligand expression was not different between KC and 4KC mice on a per-cell basis (Fig. 3D) we hypothesized that in 4KC mice, higher overall PDGF ligand levels must be found due to the increased presence of ductal cells (Fig. 2B). Indeed, Enzyme-linked immunosorbent assay (ELISA) analysis revealed significantly increased levels of the ligand PDGF-AA per gram of 4KC mouse pancreas (Fig. 5K).

### siCAFs hamper antitumor immune responses

**Fig. 6.**
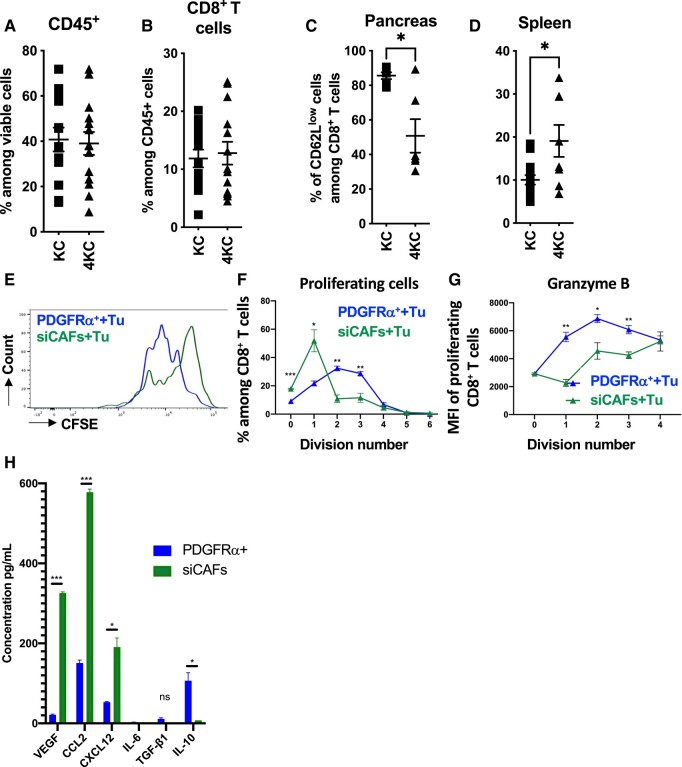
siCAFs prevent efficient T-cell activation. A and B) FACS analysis of the frequencies of CD45^+^ and CD8α^+^ cells in the pancreata of 6-week-old KC and 4KC mice. C and D) Frequencies of CD62L^low^ cells among CD45^+^/CD8α^+^/CD11c^−^ T cells in the spleen and pancreata of KC and 4KC mice. E) CFSE dilution in CD8^+^ T cells cocultured with BMDCs and CD3/CD28 activation beads in PDGFRα^+^ CAF/tumor cell (blue line) or siCAF/tumor cell-conditioned medium (green line). F and G) Proliferating (F) and GrzB (G) producing CD8^+^ T cells at the indicated division numbers after coculture with BMDCs and CD3/CD28 activation beads in PDGFRα^+^ CAF/tumor cell-conditioned (blue line) or siCAF/tumor cell-conditioned medium (green line). G) Cytokine and chemokine profiles detected in PDGRFα and siCAF in conditioned media used in E–G. A–D) Cumulative data from at least two individual experiments with three to four mice per group are shown. E–G) Representative data from two individual experiments with technical replicates are shown. PDGFRα^+^ CAFs and siCAFs were isolated from three 6-week-old 4KC mice. *P < 0.05; **P < 0.01; ***P < 0.001, unpaired t test.

Next, we investigated whether the siCAF signature associated with high tissue stiffness was also linked to immune suppression. We did not observe altered frequencies of infiltrating hematopoietic CD45^+^ cells, including CD8^+^ T cells (Fig. 6A and B), CD4^+^ T cells, CD4^+^/Foxp3^+^ regulatory T cells (Tregs), NK-p46 natural killer cells, T-cell receptor (TCR)γδ T cells or neutrophils (Fig. S6A–D), between KC and 4KC pancreata. Further analysis of CD8^+^ T cells revealed a significant reduction in T-cell activation in pancreata from 4KC mice, as indicated by the lower frequencies of CD62L^low^ (Fig. 6C). In contrast, CD8^+^ T-cell activation was significantly increased in the spleen of 4KC mice (Fig. 6D). Taken together, these data indicate that although peripheral T-cell activation appears to be efficient in 4KC mice and CD8^+^ T cells are recruited into the pancreas at similar frequencies in KC and 4KC mice, T-cell responses are hampered within the pancreatic TME.

To determine whether the two CAF populations affect CD8^+^ T-cell activation within the pancreas of 4KC mice, we isolated PDGFRα^+^ CAFs and siCAFs from 4KC mice by FACS and cocultured them with a primary tumor cell line (35) to generate CAF-conditioned medium. Next, WT CD8^+^ T cells were cocultured in the presence of bone marrow derived dendritic cells (BMDC)s and CD3/CD28 activation beads using the obtained PDGFRα^+^ CAF- or siCAF-conditioned medium. Reduced proliferation rates were detected with the siCAF-conditioned medium, as indicated by less CarboxyFluoroscein Succinimidyl Ester (CFSE) staining dilution compared with that achieved with the PDGFRα^+^ CAF-conditioned medium (Fig. 6E and F). Moreover, the production of Granzyme B (GrzB) was reduced in proliferating CD8^+^ T cells when they were cultured in the siCAF-conditioned medium (Fig. 5G). Similar results were obtained with KC PDGFRα^+^ CAF- and siCAF-conditioned medium (Fig. S6E and F). T cells cocultured in KC or 4KC PDGFRα^+^ CAF-conditioned medium showed proliferation and GrzB production similar to those cultured in control conditioned medium obtained from tumor cells alone (Fig. S6E and F). In order to determine which soluble factors could be responsible for T-cell-mediated inhibition, we performed a customized LegendPlex array for cytokines and chemokines that have been previously reported to be produced by CAFs (Fig. 6H). We detected a significant increase of Vascular endothelial growth factor (VEGF), CCL2, and CXCL12 amount siCAFs condition media compared with PDGFRα^+^ CAFs condition media. Low quantities of IL-6, TGF-β1, and IL-10 were detected in both conditions. VEGF was previously shown to have a direct immunosuppressive effect on T-cell proliferation and activation (46) suggesting that siCAF T-cell activation inhibition might be at least in part mediated by VEGF.

### PDGF neutralization reduces tumor growth and favors CD8^+^ T-cell response

**Fig. 7.**
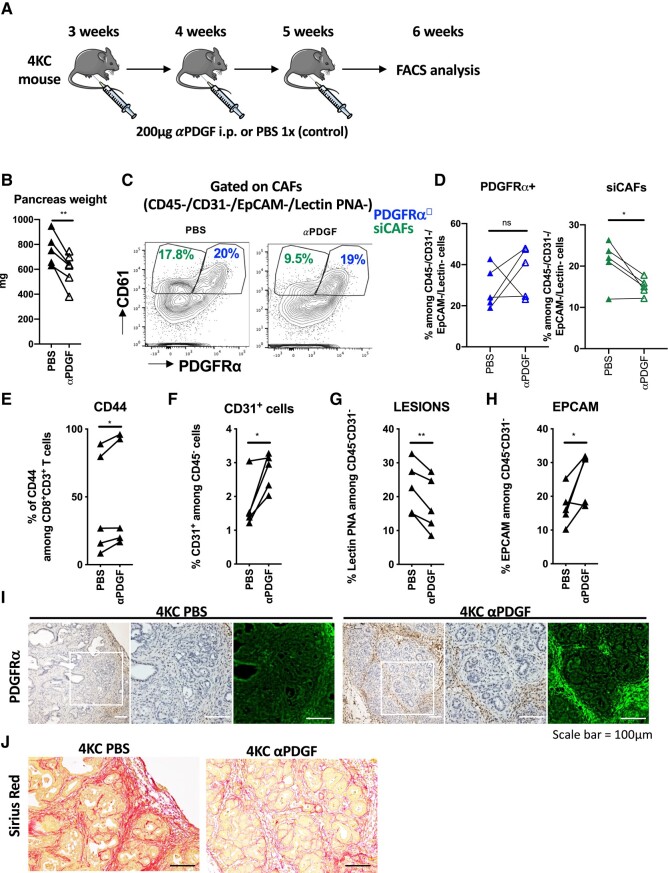
PDGF neutralization reduces stromal activation and promotes PDGFRα surface expression. A) Graphical scheme representing the treatment schedule for 4KC mice: at 3, 4, and 5 weeks of age, 200 µg of neutralizing anti-PDGF antibody diluted in 100 µL of PBS was administered ip; age-matched control mice received PBS alone. One week after the last injection, the mice were sacrificed, and harvested pancreata were subjected to FACS and IHC analyses. B) Weight (mg) of pancreata excised from 6-week-old 4KC mice. Lines connect age-matched littermates treated with the anti-PDGF antibody (white) or PBS alone (black). C) Representative FACS dot plots showing the surface expression of PDGFRα and CD61 on CAFs in pancreata from 6-week-old 4KC mice treated with the anti-PDGF antibody (right) or PBS (left). Cells were gated on viable CD45^−^CD31^−^Lectin PNA^−^EpCAM^−^CAFs. D) Frequencies of PDGFRα^+^ CAFs and siCAFs among CD45^−^CD31^−^Lectin PNA^−^EpCAM^−^CAFs in pancreata from 6-week-old 4KC mice treated with the anti-PDGF antibody or PBS. E–H) FACS analysis of the percentages of CD44^+^ among CD8^+^ T cells (E), CD31^+^ cells among CD45-cells (F), Lectin PNA^+^ (G), and EpCAM^+^ (H) cells among CD45^−^CD31^−^ cells isolated from the pancreas of 6-week-old 4KC mice treated with the anti-PDGF antibody or PBS. I) Representative images of IHC staining for PDGFRα in sections of pancreata from 6-week-old 4KC mice treated with the anti-PDGF antibody (right panel) or PBS (left panel). White squares indicate the magnified regions of each panel. The images on the right of each panel show pseudocolored PDGFRα^+^ cells (green). *P < 0.05, **P < 0.01.

Our results show that the instruction of siCAFs in 4KC mice occurs early between 1 and 1.5 months after birth and it develops in a PDGF/PDGFR signaling-dependent manner. Moreover, siCAFs prevent an efficient CD8^+^ T-cell immune response, which is a key for tumor elimination (47–49). Thus, we hypothesized that the prevention of siCAF development in 4KC mice could improve the course of the disease. Therefore, we intraperitoneally (ip) injected 4KC mice at 3, 4, and 5 weeks of age with a neutralizing polyclonal anti-PDGF antibody, which recognizes PDGF-AA, PDGF-AB, and PDGF-BB dimers. Control age-matched littermates were injected with 1× Phosphate-buffered saline (PBS). At 6 weeks of age, we analyzed pancreata collected from anti-PDGF- and PBS-injected mice (Fig. 7A). First, we observed a decrease in pancreas weight in 4KC mice injected with anti-PDGF, indicating diminished tumor growth (Fig. 7B and S7A). We next performed FACS analysis and evaluated the frequencies of siCAFs and PDGFRα^+^ CAFs among total CAFs. We observed decreased siCAFs in anti-PDGF-injected 4KC mice compared with PBS-injected 4KC mice (Fig. 7C and D). In addition, we observed an increased percentage of activated CD8^+^ T cells (CD44^+^CD8^+^ T cells) pancreas in the anti-PDGF-treated conditions (Fig. 7E). Furthermore, we observed increased CD31^+^ endothelial cells (Fig. 7F), decreased lesions (Fig. 7G), and increased EPCAM expression (Fig. 7H) in lesions, suggesting an early tumor phenotype. CD8^+^ T-cell activation, indicated by the upregulation of CD44 and CD69 as well as the downregulation of CD62L, was similar in the spleen of anti-PDGF- or PBS-injected 4KC mice (Fig. S6B–D). In addition, IHC analyses revealed the prevention of PDGFRα downregulation in pancreatic lesions by anti-PDGF injection (Fig. 7I), as well as a reduced number of advanced lesions. Furthermore, Sirius red staining showed decreased collagen fiber deposition in anti-PDGF condition (Fig. 7J). Taken together, our data show that PDGF neutralization leads to the reprogramming of the tumor environment.

### Identification of siCAFs in the human setting

**Fig. 8.**
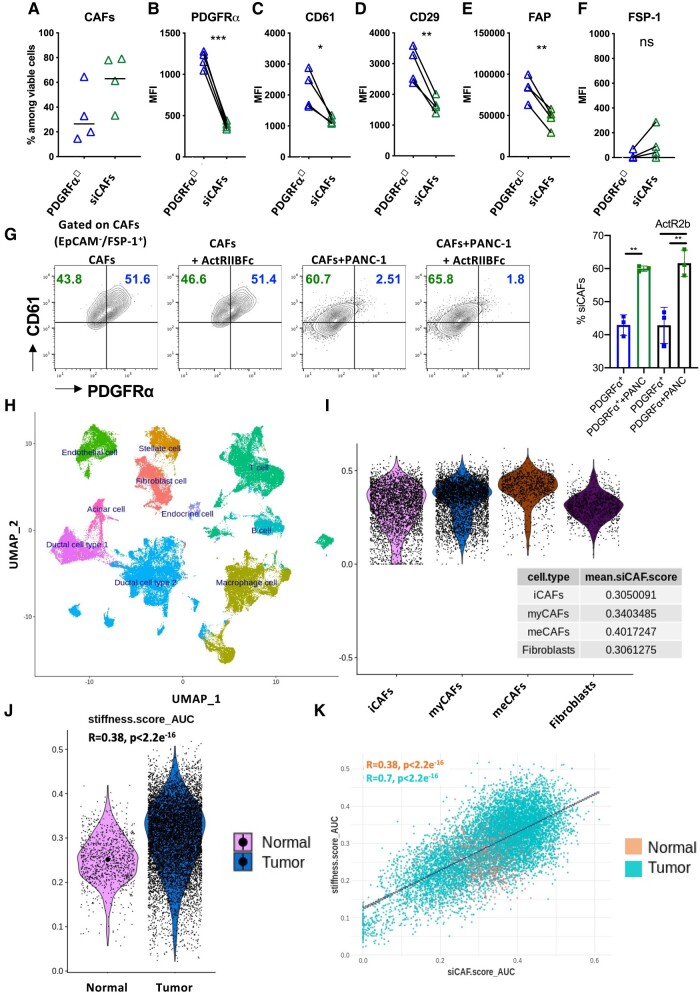
Tumor cells instruct siCAFs in human PDAC. A) FACS analysis of the frequencies of PDGFRα^+^ CAFs and siCAFs among viable cells after CAF isolation. B–F) FACS analysis of the MFIs of PDGFRα (B), CD61 (C), CD29 (D), FAP (E), and FSP1 (F) on PDGFRα^+^ CAFs and siCAFs. G) Representative FACS dot plots showing the surface expression of PDGFRα and CD61 on EpCAM^−^FSP1^+^ CAFs after coculturing with or without PANC-1 tumor cells in the absence or presence of the soluble activin A inhibitor ActRIIBFc. Frequencies of siCAFs generated in the absence or presence of ActRIIBFc. *P < 0.05; **P < 0.01; ***P < 0.001, unpaired t test (A-G). H) UMAP projection of PDAC single-cell Atlas comprising over 70 samples and >130,000 cells, most of them from cancer samples (>110,000 cells). I) siCAF signature distribution among fibroblast and described CAF subtypes. J) Previously described stiffness score distribution among tumor CAFs and normal fibroblasts. K) Correlation between the stiffness score and siCAF score.

To relate our findings to human PDAC patients, we performed FACS analysis of primary pancreatic CAFs obtained from four different donors. Similar to our mouse results, we detected an abundant siCAF population correlated with a reduced PDGFRα^+^ CAF population, indicating a differentiation-dependent connection between these two populations (Fig. 8A). Further analysis showed that the overall expression of the known CAF markers and integrins PDGFRα, CD61, CD29, FAP, and Fibroblast specific protein (FSP)-1 was significantly lower in siCAFs than in PDGFRα^+^ CAFs (Fig. 8B–F). To determine whether it is possible to reprogram siCAFs in the human setting, we performed in vitro coculture of human CAFs with human pancreatic cancer cell line PANC-1 cells, a human PDAC cell line previously described to express ALK4 (24) and respond to activin A (25), in the presence or absence of ActRIIBFc. FACS analysis showed that in the presence of PANC-1 cells, CAFs lost expression of PDGFRα which was similar to the mouse results and that the addition of ActRIIBFc did not have any effect on the amount of siCAFs. To get insight into the existence of siCAFs in human setting, we applied siCAFs signature to a scRNA dataset specific for pancreas (50). As shown in Fig. 8H, the annotated dataset includes a well-defined “fibroblast” cluster. We extracted 10,953 human fibroblasts (1,037 and 9,916 from nontumor and tumor tissues, respectively), classified these fibroblasts according to human CAF signatures (51) that distinguish iCAFs, myCAFs, and meCAF as well as normal fibroblasts and applied the 29-gene siCAF signature (38). As shown in Fig. 8I, the siCAF score was expressed in all three types of CAFs at significantly higher levels than normal fibroblasts. In order to determine whether similar association between siCAFs and tissue stiffness exists in human tissues, we took advantage of a previously published stiffness signature (Table S2) (52). This stiffness score was upregulated in tumor CAFs compared with normal fibroblasts (Fig. 8J). Moreover, siCAF and stiffness scores were significantly correlated across all PDAC CAF cells (Fig. 8K). Altogether, these data suggest for the first time that paracrine signaling between tumor cells and CAFs is able to induce siCAFs phenotype independent of the genetic background.

## Discussion

In this study, we show for the first time that targeting PDGF signaling through a ligand trap approach is able to inhibit tumor progression by reprogramming the activation status of the CAFs. Despite the previously described heterogeneity of CAF populations (19–21), we showed here that PDGFRα and CD61 were able to define two activation states reflecting the stiffness of the TME and that physical constraint was able to remodel the immune response outcome and the consequent tumor progression.

Enhancement of intrinsic PDGFR signaling induced by mutational activation of PDGRFA, but not PDGRFB, systemically induced fibroblastic hyperplasia and increased ECM deposition similar to that observed in collagen diseases (53). PDGFRA point mutations (gain of function) were reported to be associated with gastrointestinal tumors (54, 55). The secretion of PDGFs by platelets and their effects on fibroblasts play a role in wound healing (56). Exogenous PDGF-BB accelerates ulcer healing in diabetic patients (57). High expression of *PDGFA* has been reported to predict a poor prognosis in esophageal squamous cell carcinoma (58). Transgenic mice overexpressing PDGFA have been shown to display severe fibrotic reactions in the heart supporting the crucial role of this ligand in ECM deposition (59). PDGF-AA binds primarily to PDGFRα, while PDGF-AB and PDGF-BB bind to PDGFRα as well as other receptor subtypes, such as PDGFRβ (60). Our results are supported by previous reports showing that stromal constitutive activation of PDGFRα (by using mice that harbor a mutation in the activation loop of PDGFRα tyrosine kinase domain) in the mammary stromal fibroblast compartment led to fibrosis and concomitant increase in collagen deposition (61). We demonstrated here that continuous binding of PDGF-AA to PDGFRα induced receptor downregulation, leading to the emergence of siCAFs. Our data are in accordance with previous findings in vascular smooth muscle cells showing that PDGFRα protein was decreased on stiff substrates, indicating internalization and degradation through ligand bound to the receptor (62). Furthermore, previous work highlighted that in PDAC, PDGRFA expression by CAFs is associated with an inflammatory phenotype in iCAFs and FB1 CAFs (20, 63). The expression analysis of genes encoding CAF markers other than PDGFRA and PDGFRB (64, 65) showed that FSP1 expression was significantly increased on PDGFRα^+^ CAFs from 4KC mice (stiff conditions). This finding was validated by the high expression of FSP1 observed in a human setting, suggesting that FSP-1 might be a universal bona fide marker of activated CAFs. The expression of integrins, such as CD29/ITGB1 (66, 67) or CD61/ITGB3 (68), on the surface of CAFs has been previously described. Here, we show that CD29 and CD61 expression was downregulated on siCAFs in both mouse and human settings, suggesting an active role in signaling. Previous work from our group showed that βig-h3/TGF-βi was able to bind CD61 and phosphorylate Lck at Y505 (35, 36) and Focal adhesion kinase (FAK) (69). We reported that βig-h3 was highly expressed in stiff conditions and that the use of an anti-βig-h3 antibody was able to reduce TME stiffness (35, 69), suggesting that targeting both PDGF-AA and βig-h3 might represent a promising therapeutic combination.

Herein, the pro- and anti-inflammatory properties of CAFs are demonstrated to be highly dependent on intratumoral location and less dependent on origin. We demonstrated that PDGFRα^+^ CAFs had no potential to inhibit a T-cell response in vitro under the loose condition (KC condition). Under stiff conditions, this population had less inhibitory potential than siCAFs, highlighting the importance of the mechanical properties of the tissue in the education of CAFs. Several publications have demonstrated that these cells are important for sensing mechanical changes in tissues not only at homeostasis but also in pathological conditions, i.e. fibrosis. The important finding of this study is that despite the origin of the cells, the mechanical constraint together with the secretion of soluble factors breaks the heterogeneity of CAFs into a binary classification according to their “microenvironment sensing,” namely, PDGRFα^+^ CAFs and siCAFs. This creates the option of targeting a pathway through a ligand trap approach that would reprogram siCAFs into PDGFRα^+^ CAFs rather than targeting a particular surface marker. This might also be beneficial since the elimination of CAFs by antibody-dependent cytotoxicity has been shown to be deleterious in PDAC (clinical trial failure).

A hallmark of the pathogenesis of solid cancers such as PDAC escapes from efficient antitumor immune responses. It has been demonstrated that the collagenous TME can restrain infiltrating CD8^+^ T cells from accessing tumor cells (70, 71). Additionally, the cytotoxic activity of tumor-infiltrating lymphocytes can be reduced by insufficient T-cell priming (72). CD8^+^ T-cell exclusion from the tumor bed has been proven to be a key element in the antitumor response (48). We show here that early mechanical and paracrine education through PDGF ligands (PDGF-AA in particular) can skew an effective antitumor response in situ. Although we identified potent immune priming of CD4^+^ and CD8^+^ T-cell responses in the spleen, the number of activated CD8^+^ T cells in the stiff pancreas was diminished compared with that in the loose pancreas, indicating that local tissue mechanics are a key element in the outcome of the immune response. Further studies combining spatial detection of activated CD8^+^ T cells and tissue rigidity would provide indications of the outcome of immune checkpoint therapy. Interestingly, we also observed an increase in CD31 expression in PDGF-depleted conditions, suggesting that an increase in modified interactions at the level of blood vessels might involve other partners that prevent an efficient T-cell response (i.e. MDSCs or macrophages). Further investigation linking the availability of PDGF-AA to angiogenesis should shed new light on this additional mechanism of action.

Based on our data, we established a model of the mechanism of action. As tumors evolve, they proliferate, produce PDGF ligands, and instruct CAFs via a paracrine effect. PDGFRα^+^ CAFs become siCAFs capable of inhibiting T-cell responses in situ. By using a PDGF-AA ligand trap approach, neoplastic tissue homeostasis can be restored. Neutralization of the PDGF-AA leads to PDGRFα^+^ CAF maintenance associated with soft conditions and an efficient T-cell response. Our study provides support for the translational potential of using a PDGF ligand trap strategy.

## Supplementary Material

pgad405_Supplementary_Data

## Data Availability

All data will be publicly released. GEO Accession: GSE231348. https://www.ncbi.nlm.nih.gov/geo/query/acc.cgi?acc=GSE231348
