# Supplementary material for: Stiffness-induced cancer-associated fibroblasts are responsible for immunosuppression in a platelet-derived growth factor ligand-dependent manner
Source: PNAS Nexus. 2023 Dec 18;2(12):pgad405. doi: 10.1093/pnasnexus/pgad405 (PMC10727001; doi:10.1093/pnasnexus/pgad405)
Supplement: pgad405_Supplementary_Data [file pgad405_supplementary_data.zip › Supplementary Materiel7112023.pdf]

Supplementary Information

Immunohistochemistry (IHC) and immunofluorescence (IF)

Formalin-fixed paraffin-embedded (FFPE) mouse pancreata were sectioned at 4 □m. FFPE samples were rehydrated by heating slides at 55°C for 30 minutes and successive baths of xylene and ethanol (100%, 95%, 70%). In sections submitted for IHC, endogenous peroxidase was inactivated with 3% H_2O_2. IHC and IF staining procedures were performed after heat-induced epitope retrieval using an antigen-unmasking solution (Vector Laboratories, US). The sections were incubated with primary antibodies overnight at 4°C in a wet chamber. Next, the sections were incubated with biotinylated (IHC) or fluorochrome-conjugated (IF) secondary antibodies for 1 h at room temperature (RT). IHC staining was visualized using 3,3'-diaminobenzidine (DAB kit; Vector Laboratories, UK), and the sections were counterstained with hematoxylin. IF sections were counterstained by mounting with Vectashield mounting medium with DAPI. Hematoxylin & eosin (H&E) staining was performed using a standard protocol. IHC and H&E staining was imaged using a Carl Zeiss microscope Axioscan 7. IF images were acquired on a Carl Zeiss LSM 880 microscope. FIJI imaging software was used for visualization and/or analysis of IHC, IF, and H&E staining.

Flow cytometric analysis

Single-cell suspensions were stained with fluorochrome-labeled antibodies for 20 minutes at 4°C. Fixable viability dye (BioLegend) or DAPI staining was performed to exclude dead cells from the analysis. The stained cells were fixed directly by incubation in FACS buffer supplemented with 4% paraformaldehyde (PFA) for 20 minutes at 4°C, or intracellular staining was performed using the Fixation/Permeabilization Kit (BD Biosciences). No fixation/permeabilization was performed if the cells were subjected to FACS sorting. Stained cells were acquired on a BD Fortessa flow cytometer (BD Biosciences) for FACS analysis. For CAF and ductal cell isolation, cells were acquired on a BD FACSAria (BD Biosciences), and the sorted cells were collected in DMEM containing 10% (functional assays) or 20% fetal calf serum (FCS) (RNA isolation). FACS data were analyzed using FlowJo software (TreeStar).
A list of the antibodies used for flow cytometry can be found in the Supplementary Methods.

Supplemental list of FACS antibodies

The following antibodies and reagents were used for flow cytometric analysis and FACS sorting of cells freshly isolated from mouse pancreata or spleens:

anti-CD4-BV605 or anti-CD4-PE (RM4-5); anti-CD8α-PE-Cy7 (53-6.7); anti-CD11c-BV510 (N418); anti-CD29-PerCP-Cy5.5 (HMβ1-1); anti-CD31-BV421 (390); anti-CD44-AF700 (IM7); anti-CD45-BV421, anti-CD45-APC-Cy7 or anti-CD45-BV650 (30-F11); anti-CD61-PE-Cy7 (2C9.g2); anti-CD62L-PE or anti-CD62L-BV510 (MEL-14); anti-CD69-BV785 (H1.2F3); anti-CD326/EpCAM-FITC or anti-CD327/EpCAM-BV421 (G8.8); anti-CD355/NKp46-BV421 (29.A1.4); anti-Foxp3-AF647 (MF-14); anti-Granzyme B-AF647 (GB11); and anti-Ly6G-AF700 (1A8) were obtained from BioLegend. Anti-αSMA-eF660 (1A4), anti-CD11c-AF700 (N418), anti-CD31-SuperBright 645 (390), anti-CD45-APC (30-F11), anti-FSP1/S100-Biotin (4C4.9), anti-GFAP-AF488 (GA5), anti-PDGFRα/CD140a-APC-eF780 or anti-PDGRα/CD140a-SuperBright 600 (APA5), anti-PDGFRβ/CD140b-SuperBright780 (APB5), and anti-TCRγδ-APC (eBioGL3) were obtained from ThermoFisher. Anti-FAP1-AF488 (Polyclonal) was obtained from Bioss Antibodies/CliniSciences, anti-CD44-PE (KM18) was obtained from Immuno Tools, and anti-CD69-PerCP-Cy5.5 (H1.2F3) was obtained from BD Biosciences. Anti-Lectin PNA-AF568 or anti-Lectin PNA-AF647 was obtained from ThermoFisher. Streptavidin-BV510 was obtained from BioLegend.

Antibodies against the following markers were used for flow cytometric analysis of primary PDAC patient-derived CAFs:

CD29, CD61, FAP1, PDGFRα/CD140a, and PDGFRβ/CD140b.

Supplemental list of IHC and IF antibodies

The following antibodies were used for IHC or IF staining:

Purified anti-CD61/ITGβ3 (EPR20825) obtained from Abcam and anti-PDGFRα/CD140a (16A1) obtained from BioLegend were revealed with biotinylated horse-α-rabbit and goat-α-mouse secondary antibodies, respectively. Purified anti-CK19 (TROMA-III) obtained from Developmental Studies Hybridoma Bank (DSHB) and anti-αSMA (polyclonal) obtained from GeneTex were revealed with donkey α-rat-AF488 and donkey α-rabbit-Cy3 secondary antibodies, respectively.

AFM: protocol application

The mechanical properties of pancreatic tissue areas were determined by AFM as described previously (34). Briefly, in AFM, the tip of a cantilever is pushed against sample tissue, and its deflection is monitored. Based on the stiffness constant of the cantilever, the deflection indicates the resisting force of the sample (34). The applied protocol allows the measurement of tissue stiffness very locally in a minimally invasive manner by deforming the sample down to a depth of 100 nm.

The stiffness patterns of different regions within a pancreatic lesion (stromal compartment and tumor cells) were determined at high resolution by applying quantitative nanomechanical mapping and force volume protocols (Bruker). In these protocols, the AFM probe oscillates at a low frequency while horizontally scanning the sample and generating a force curve each time the probe contacts the sample. The elastic modulus reflecting the stiffness of the sample is extracted from each curve by applying the Sneddon (Hertz) model, resulting in a 2D stiffness map in which each pixel represents one force curve.

Cell isolation from mouse tissues

Excised mouse pancreata were washed in phosphate-buffered saline (PBS) and minced into small fragments, followed by incubation in a collagenase solution (1 mg/ml collagenase P obtained from Sigma-Merck in HBSS) at 37□C for 20 minutes. A single-pancreatic cell suspension was obtained by sequentially filtering the digested tissue through a 100-µm cell strainer followed by a 70-µm cell strainer. Spleens were homogenized by filtration through a 100-µm cell strainer to obtain single-cell suspensions. Red blood cells were lysed using NH_4Cl lysis buffer.

scRNAseq: quality control and data analysis

FACS-purified CAFs and ductal tumor cells from a pool of five KC or 4KC mice were partitioned into nanoliter-scale gel bead-in-emulsions (GEMs) with the Chromium Single Cell Controller (10x Genomics) at the in-house Single Cell Platform (CLB/CRCL). After cell encapsulation and barcoding, library preparation followed the standard 10x Genomics 3'scRNAseq protocol comprising reverse transcription, amplification, and indexing. Sequencing was performed using a NovaSeq Illumina device (Illumina). Illumina bcl files were basecalled, demultiplexed and aligned to the mouse mm10 genome using CellRanger software (10x Genomics).

Count data (filtered barcode matrices) were obtained with CellRanger (10xGenomics). All downstream analyses were performed using R/Bioconductor/CRAN packages, R version 4.2.2 (2022-11-10) [https://cran.r-project.org/; http://www.bioconductor.org/; https://cran.r-project.org/] on a Linux platform (x86_64-pc-linux-gnu [64-bit]). Filtered barcoded matrices were used to create Seurat objects (81) for each condition that were subsequently merged (package 'Seurat' v.4.1.1).

A total of 8878 cells (3638 4KC CAFs, 3664 KC CAFs, 901 4KC Ducts, and 675 KC Ducts) remained after filtering for quality parameters (number of features per cell between 1000 and 6000, fraction of mitochondrial genes < 10%). The Seurat SCTransform function was used to

simultaneously normalize, identify variable features and scale the data. Following dimension reduction with principal component analysis (PCA), the first 30 dimensions were used to construct a shared nearest neighbor (SNN) graph using the FindNeighbors function. Clusters were identified with a resolution of 0.1 and projected in two-dimensional plots using UMAP [arXiv: 1802.03426v3]. The markers of each cluster and DEGs in pairwise comparisons were identified using the FindAllMarkers and FindMarkers functions, respectively (main parameters: only.pos = F, min.pct = 0.25, and logfc.threshold = 0.25), with an adjusted p value threshold of 0.05. Fibroblasts (CAFs) were identified and re-clustered with a higher resolution (0.5). Known CAF markers(23, 24) were used to score each cell according to their myofibroblastic (myCAFs), inflammatory (iCAFs), and antigen-presenting (apCAFs) signatures, using the AUCell package (v.1.18.1). Pathway analyses were performed with the enrichment functions of the 'ClusterProfiler' package (v.4.7.1). Pathway scores were estimated with the Seurat AddModuleScore function using 100 control features after downloading relevant pathways with the R packages enrichR v.3.1 (82, 83) and pathfindR v.1.6.3 (84).

'SingleCellSignalR' v.1.8.0 was used to study cell interaction networks on Seurat preprocessed data, using the major cell type labels (i.e. ducts, CAFs), independently for the two study conditions (i.e. KC and 4KC). All receptor:ligand analyses were done in "paracrine" mode and visualized with the chord plot and heatmap functions of the same package. siCAF signature using the AUCell method to calculate a score for every single cell (46).

CAF differentiation assay: coculture of isolated PSCs and acinar cells

PSCs were isolated from wild-type (WT) C57BL/6 mice as previously described (17, 23). Briefly, a single-pancreatic cell suspension was resuspended in 9 ml of GBSS containing 0.3% BSA and 43.75% Histodenz (Sigma-Merck), placed into a 15-ml conical tube and overlaid with 6 ml of GBSS containing 0.3% BSA. After gradient centrifugation, the cells within the gray band just above the interface between the GBSS and Histodenz layers were harvested and used for CAF differentiation.

Acinar cells (Acs) were isolated from KC and 4KC pancreata after digestion in a collagenase/soybean trypsin inhibitor solution (1 mg/ml collagenase P and 25 µg/ml soybean trypsin inhibitor, both obtained from Sigma-Merck, in HBSS).

PSCs and acinar cells were labeled using a CellTrace-CFSE or CellTrace-Violet proliferation kit (Invitrogen), respectively, and cocultured in DMEM (Gibco) containing 10% FCS, penicillin/streptomycin, and 0.2 mg/ml soybean trypsin inhibitor (Sigma-Merck) at a ratio of

1:2 in 24-well plates equipped with discs made of rat tail collagen (Sigma-Merck). For certain conditions, the activin A inhibitor ActRIIBFc (gift from Olli Ritvos, Helsinki, Finland) was added at a final concentration of 0.5 µg/ml. After six days, the cells and collagen plates were recovered, and FACS analysis was performed to evaluate the differentiation of WT PSCs into CAFs.

Mouse cells lines

The isolation and culture of cells were performed using a protocol adapted from previously published protocol (35). Tumor primary cell line 4KC was obtained from the pancreas of 2.5-months old 4KC mice using the same protocol. After several passages, the cells were infected with a lentivector expressing H2B GFP as previously described (36). Immortalized mouse pancreatic stellate cells (iPSCs) were obtained from Tuveson DA (23).

Human CAF generation

Small pancreatic tissue blocks were obtained from patients with resectable PDAC during pancreatic surgery. The experimental procedure relating to the use of patient-derived pancreatic tumor pieces was performed after approval by the South Mediterranean Personal Protection Committee under reference 2011-A01439-32. Primary CAFs were isolated as previously described (37). Briefly, tumors were cut into small pieces (1 mm^3) using a razor blade. The tissue pieces were dissociated using the Tumor Dissociation Kit (Miltenyi Biotec; 130-095-929) according to the manufacturer's recommendations. The cells were then resuspended, passed through a cell strainer (100 µM), and plated. Primary CAFs were used between passages 4 and 8. Primary CAF features were verified by flow cytometry with positive α-SMA and FAP staining. Immortalized CAFs were generated from primary CAFs of limited passage via retrovirus-mediated expression of human telomerase reverse transcriptase (hTERT).

Human primary CAFs were cultured in Dulbecco's modified Eagle's medium (DMEM)/F-12 supplemented with 10% fetal bovine serum (Biosera FB-1001/500), 2 mmol/l l-glutamine (Invitrogen; 25030-024), 1% antibiotic-antimycotic (Invitrogen; 15240-062), and 0.5% sodium pyruvate (Invitrogen; 11360-039). Human immortalized CAFs were cultured in DMEM/F-12 supplemented with 10% fetal bovine serum and 1% antibiotic-antimycotic. The pancreatic cancer cell line PANC-1 was obtained from ATCC and cultured in DMEM GlutaMAX (Gibco 10566016) supplemented with 10% fetal bovine serum and 1% antibiotic-antimycotic. For coculture experimental conditions, primary CAF medium was used. Cells were authenticated through an STR profile report (LGC Standard) and confirmed to be mycoplasma free (Lonza, LT07-318).

PANC-1 cells were plated 24 h before coculture with CAFs in triplicate for each experimental condition and treated with 0.5 µg/ml ActRIIbFc inhibitor. The following day, human primary or immortalized CAFs were plated in monoculture or coculture according to the experimental conditions at a 2:1 ratio with PANC-1 cells and were treated with 0.5 µg/ml ActRIIbFc inhibitor. Half of the cell culture medium was refreshed every 48 h with the addition of 0.5 µg/ml ActRIIbFc inhibitor until day 6 of culture. Cells were detached using StemPro Accutase cell dissociation reagent (Gibco A1110501) and washed once with PBS. Samples were resuspended in FACS buffer (0.5% BSA and 2 mM EDTA in PBS) and surface stained with the following fluorochrome-coupled antibodies: anti-CD61 (BioLegend 370010), anti-CD140a/PDGFRα (BD Biosciences 742666), anti-FAP (R&D Biotechne FAB3715R), and anti-EpCAM (BioLegend 324204). Intracellular staining using anti-α-SMA (R&D Biotechne IC1420P) and anti-FSP-1 (BioLegend 370010) antibodies was performed following fixation and permeabilization with BD Cytofix/Cytoperm (554714). Samples were analyzed on a BD LSRFortessa X20 cell analyzer.

PDGFRα tyrosine phosphorylation and Western blot

iPSCs were seeded onto 6 wells plate (4.0 x10^5 cells per wells). Treatments of various lengths (10min, 30min, 2h) were done with 4KC conditioned medium or mouse recombinant PDGF-AA protein (Biolegend,776304) at 50ng/mL. For proteasome inhibition, cells were starved and treated with the proteasome inhibitor MG-132 (Sigma Aldrich, M8699-1MG) at 10µM for 12h. Cell extracts were prepared from cultured cells lysed by scratching at 4°C in 80uL RIPA (ThermoFisher, 89900) supplemented with protease (Roche, 04693159001) and phosphatase inhibitors (Roche, 04906837001). Obtained lysates were sonicated and centrifugated at 13,000g for 15min at 4°C. Equal amount of proteins were separated by SDS-PAGE then transferred onto Immune-Blot PVDF membrane. Membranes were incubated in blocking buffer containing 5% milk or Bovine Serum Albumine (BSA) (Sigma Aldrich, A2153-100G) in Tris Buffered Saline/Tween 20 (TBST). The blots were then probed overnight at 4°C with the appropriate primary antibodies. The membranes were revealed with the appropriate secondary antibodies for 1h at RT. Detection was performed by enhanced chemiluminescence using Pierce™ ECL Western Blotting Substrate (Thermo Scientific, 32106) according to the manufacturer's protocol. Tubulin was used as a loading control. Antibodies and dilutions were as follows: anti-p-Ty754 Ab (Thermo Fisher, TF441008G), 1:1000; anti-PDGFRα (Cell Signalling Technology, 3174S),1:1000; anti-Tubulin (GenTex, GTX628802), 1:1000.Secondary HRP-

conjugated anti-rabbit Ab (Jackson Immuno Research, 711-035-152) or anti-mouse Ab (Jackson Immuno Research, 715-035-150).

CAF and 4KC-conditioned medium

For the generation of conditioned medium, CAF subpopulations isolated by FACS were cocultured with cells from the KIC tumor cell line (35) at a ratio of 1:1 in DMEM containing 10% FCS. After a 48-h incubation at 37ºC and 5% CO_2, the supernatants were collected and stored at -20°C until further use. For the generation of conditioned medium, 4KC cells (1.5 x10^4 cells per wells) were seeded onto 6 wells plate in DMEM containing 10% FCS. After 7 days of incubation at 37°C and 5% CO2, the supernatants were collected and stored at -20°C until further use.

Bone marrow-derived dendritic cell (BMDC) generation

To generate bone marrow-derived monocytes, bone marrow cell suspensions were isolated by flushing the femurs and tibias of 8- to 12-week-old C57BL/6 mice (Charles River) with DMEM containing 10% FCS as previously described (36). Cell aggregates were dislodged by passing the suspension through a 70-µm cell strainer. Lysis of red blood cells was performed with ammonium-chloride-potassium (AKC) lysis buffer. The obtained cells were incubated for 6 days at 37ºC and 5% CO_2, and every other day, fresh DMEM containing 10% FCS and GM-CSF was added.

T-cell proliferation assay

Spleen and lymph nodes from 8 to 12-weeks old C57BL/6 mice were harvested, mechanically dissociated and cell suspension was incubated with anti-CD8α magnetic beads following manufacturer's protocol (Miltenyi Biotec). MACS-purified $CD8^+$ T cells were labeled with a CellTrace-CFSE proliferation kit (Life Technologies) according to the manufacturer's protocol. The CSFE-labeled $CD8^+$ T cells were cultured for 2 days in the presence of BMDCs (T cells:BMDCs=16:1). Mouse T-Cell-Activator CD3/CD28 Dynabeads (Gibco) were added to the coculture (T cells:Beads=1:1). The proliferation of the $CD8^+$ T cells was evaluated at the end of the culture period by analyzing CSFE dilution using flow cytometry.

Enzyme-linked immunosorbent assay (ELISA)

To determine the amount of PDGF-AA in tissue samples, freshly excised mouse pancreata were weighed and finely minced. Then, 500 µl of 1x PBS was added to the tissue fragments of each pancreas and thoroughly mixed. After centrifugation for 5 minutes at 300 g, the supernatant was collected, and a mouse PDGF AA ELISA kit obtained from Abcam was used according to the manufacturer's protocol.
