# Supplementary material for: Stiffness-induced cancer-associated fibroblasts are responsible for immunosuppression in a platelet-derived growth factor ligand-dependent manner
Source: PNAS Nexus. 2023 Dec 18;2(12):pgad405. doi: 10.1093/pnasnexus/pgad405 (PMC10727001; doi:10.1093/pnasnexus/pgad405)
Supplement: pgad405_Supplementary_Data [file pgad405_supplementary_data.zip › GamradtealsSuppFiguresfinal.pdf]

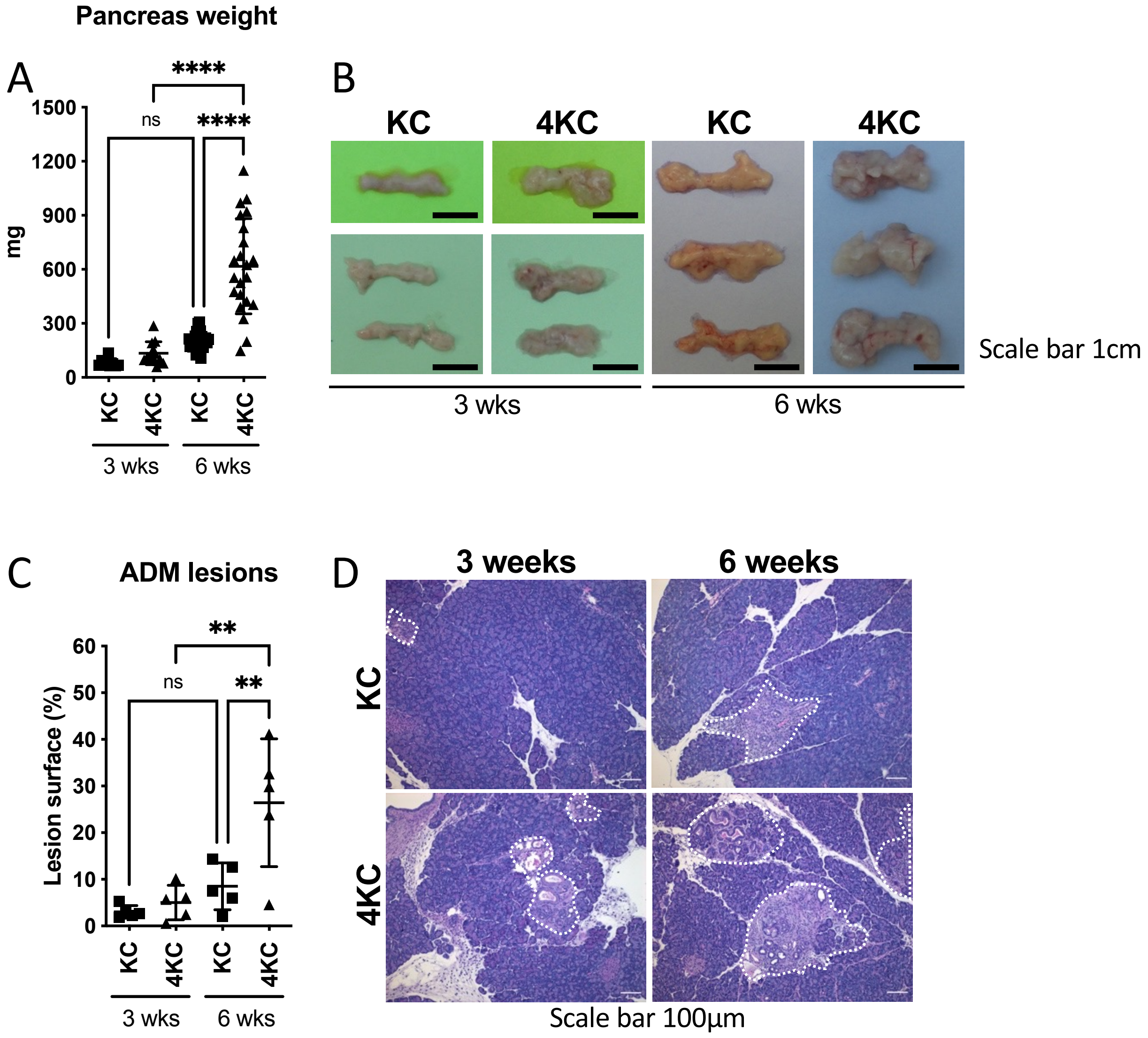

Supplementary Fig. 1

Supplementary Figure 1: ALK4 signaling disruption in tumor cells promotes tumor progression and stromal development.

(A) Weight (mg) of pancreata excised from three- or six-week-old KC and 4KC mice. Cumulative data from three individual experiments with 3-5 mice per group are shown. (B) Representative photographs of pancreata from three- (left panel) or six-week-old (right panel) KC and 4KC mice. (C) Quantification of the lesional pancreatic surface of three- or six-week-old KC and 4KC mice (bottom). Representative data from two individual experiments with 5 mice per group are shown. (D) Representative photographs of pancreata from three- (left panel) or six-week-old (right panel) KC (top) and 4KC mice (bottom); dashed lines mark representative lesions included in the quantification. (A and C) The mean values □ SEMs are displayed. **p <0.01; ****p <0.0001.

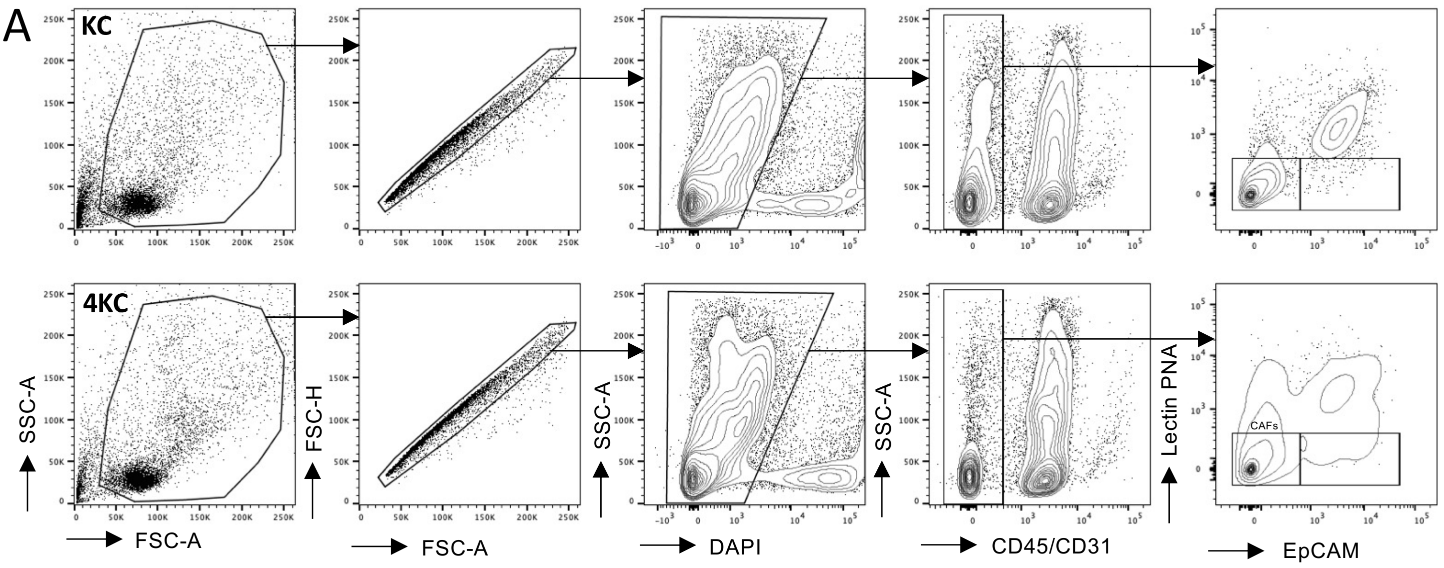

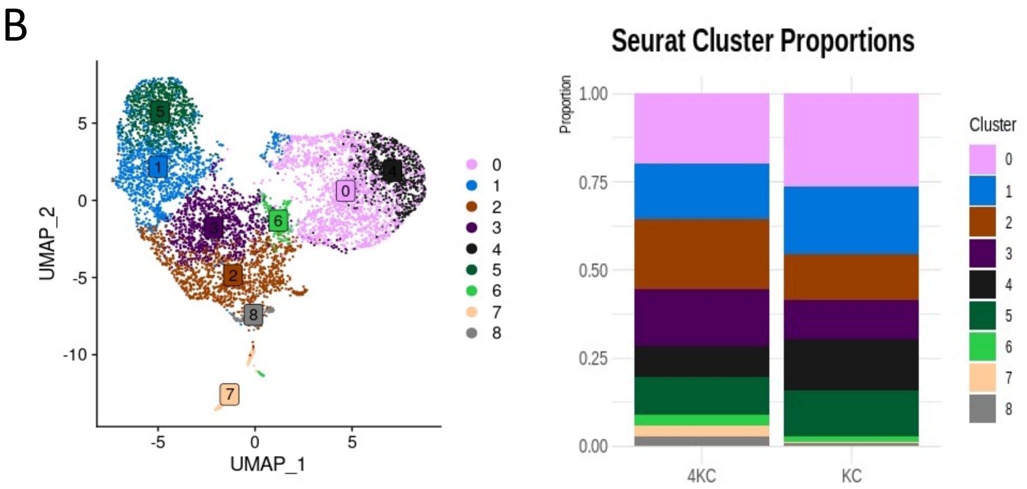

	4KC	KC
**0**	19.68	26.23
**1**	15.72	19.30
**2**	20.07	12.91
**3**	16.22	11.30
**4**	8.77	14.52
**5**	10.45	12.80
**6**	3.08	1.58
**7**	3.24	0.57
**8**	2.78	0.79

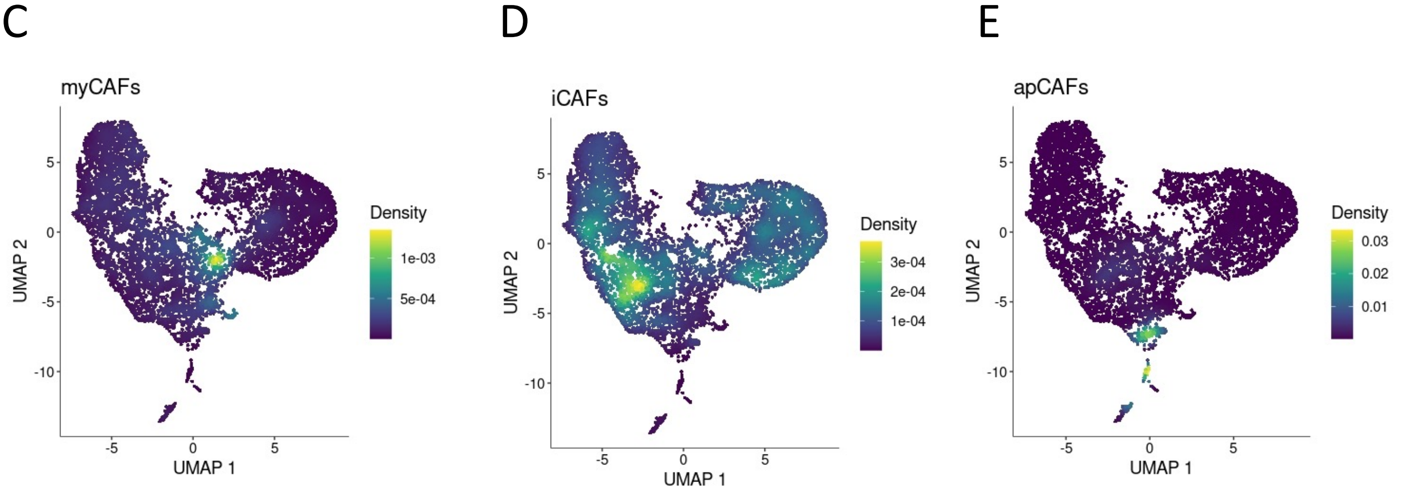

Supplementary Fig. 2

F

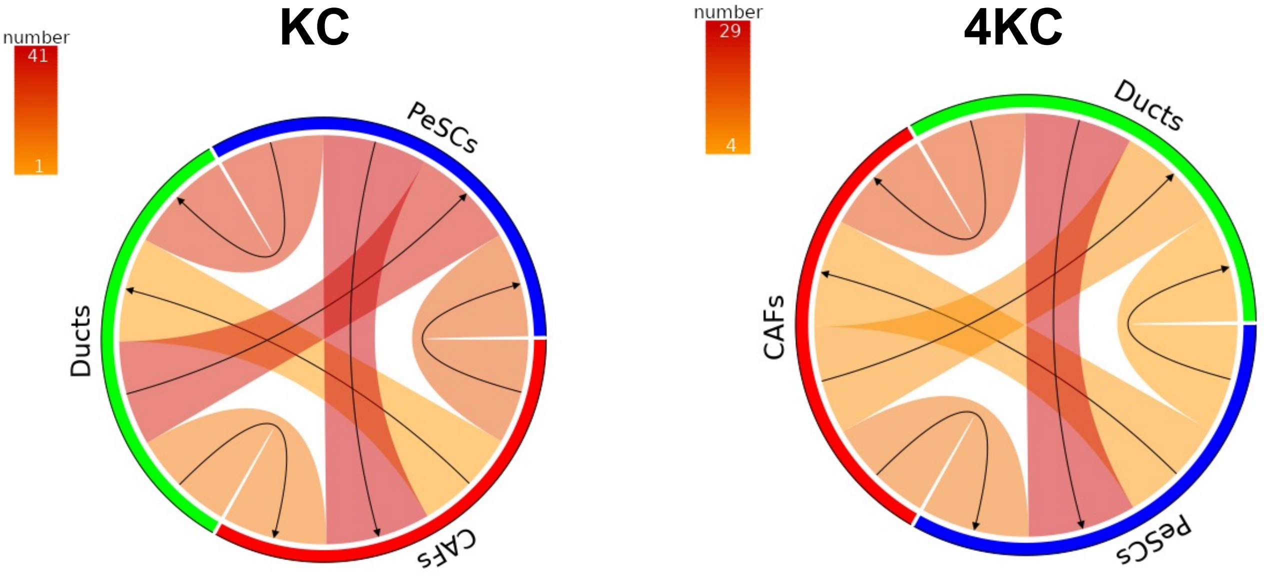

G

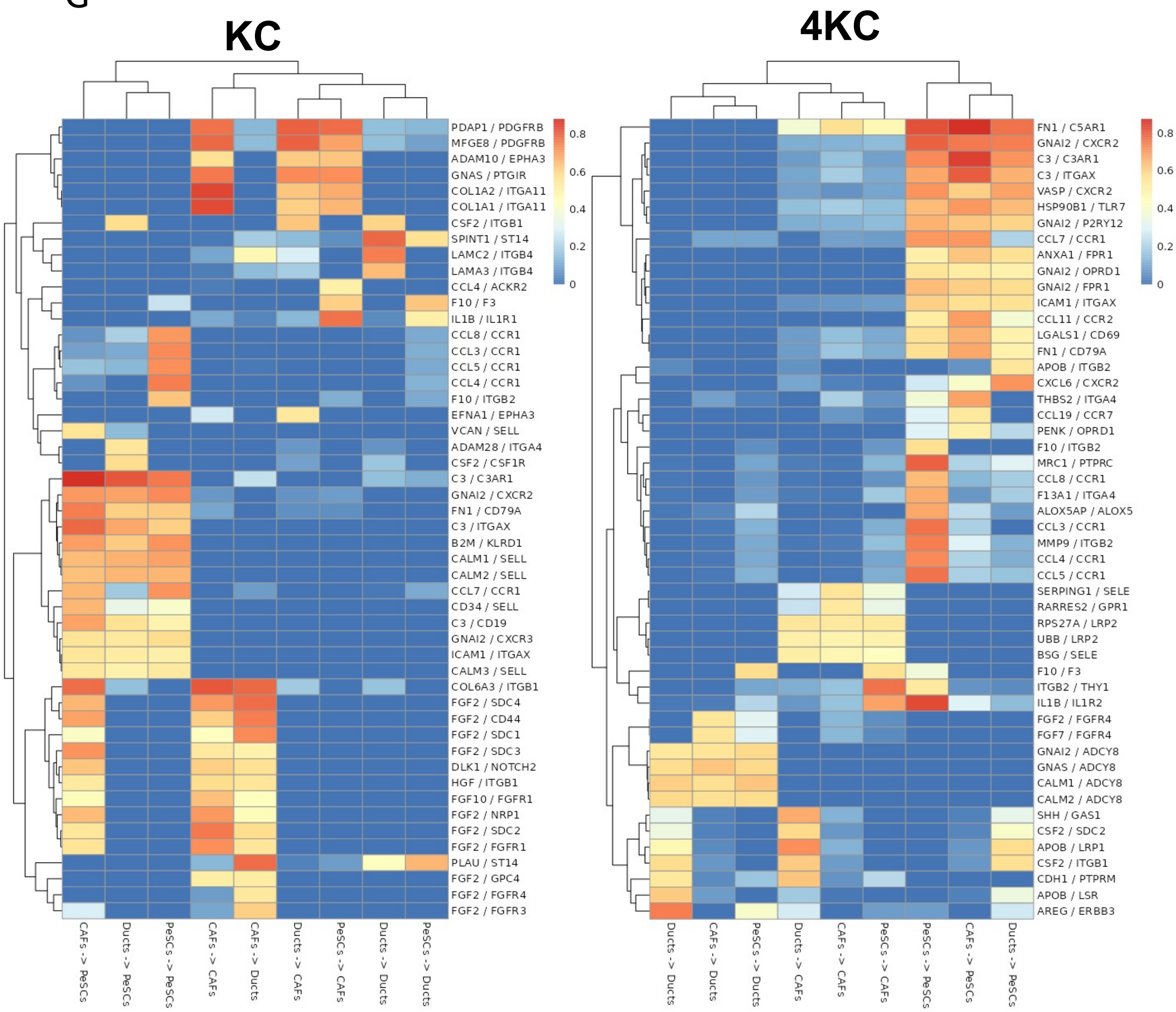

Supplementary Fig. 2

H

KC

ligand	receptor	interaction.type	LRscore
CAFs.Dcn	Ducts.Erbb4	paracrine	0,720650548
Ducts.Gnas	CAFs.Ptgir	paracrine	0,761025074
Ducts.Gnai2	CAFs.S1pr3	paracrine	0,744468335
Ducts.Col1a2	CAFs.Itga11	paracrine	0,660710975
Ducts.Adam10	CAFs.Epha3	paracrine	0,653949874
Ducts.Col1a1	CAFs.Itga11	paracrine	0,644123049
Ducts.Efna4	CAFs.Epha3	paracrine	0,581752451
Ducts.Efnb2	CAFs.Epha3	paracrine	0,580854742
Ducts.Efna1	CAFs.Epha3	paracrine	0,571991179
Ducts.Efna5	CAFs.Epha3	paracrine	0,532367775
Ducts.Liph	CAFs.Lpar4	paracrine	0,521164091
Ducts.Il1rn	CAFs.Il1r2	paracrine	0,518848794

4KC

ligand	receptor	interaction.type	LRscore
CAFs.Gnas	Ducts.Adcy8	paracrine	0,658399288
CAFs.Dcn	Ducts.Erbb4	paracrine	0,656188423
CAFs.Calm1	Ducts.Adcy8	paracrine	0,607756525
CAFs.Calm2	Ducts.Adcy8	paracrine	0,605529211
CAFs.Gnai2	Ducts.Adcy8	paracrine	0,587255586
CAFs.Calm3	Ducts.Adcy8	paracrine	0,512820559
Ducts.Mfge8	CAFs.Pdgfrb	paracrine	0,817461691
Ducts.Pdap1	CAFs.Pdgfrb	paracrine	0,808549034
Ducts.Calm1	CAFs.Pde1a	paracrine	0,796738991
Ducts.Calm2	CAFs.Pde1a	paracrine	0,783743393
Ducts.Pdgfa	CAFs.Pdgfrb	paracrine	0,747897849
Ducts.Gnai2	CAFs.S1pr3	paracrine	0,746085913
Ducts.Pdgfb	CAFs.Pdgfrb	paracrine	0,727260299
Ducts.Pdgfc	CAFs.Pdgfrb	paracrine	0,684227532
Ducts.Calm3	CAFs.Pde1a	paracrine	0,661038708
Ducts.Pdgfd	CAFs.Pdgfrb	paracrine	0,63106737
Ducts.App	CAFs.Ngfr	paracrine	0,610311843
Ducts.Rps27a	CAFs.Lrp2	paracrine	0,582174411
Ducts.Rtn4	CAFs.Ngfr	paracrine	0,579450283
Ducts.Liph	CAFs.Lpar4	paracrine	0,547120748
Ducts.Bsg	CAFs.Sele	paracrine	0,545015849
Ducts.Ubb	CAFs.Lrp2	paracrine	0,53970867
Ducts.Pyy	CAFs.Fap	paracrine	0,538041911
Ducts.Hspa8	CAFs.Lrp2	paracrine	0,505137277

Supplementary Fig. 2

Supplementary Figure 2: Single-cell RNA sequencing of pancreatic CAFs and ducts.

(A) Gating strategy for CAF and ductal cell isolation by FACS sorting. Briefly, after enzymatic/physical dissociation, the cells obtained from the pancreata harvested from five KC (top row) or five 4KC (bottom row) mice were stained with fluorochrome-labeled antibodies and subjected to FACS sorting. After exclusion of cell doublets, DAPI$^+$ dead cells, CD45$^+$ hematopoietic cells, and CD31$^+$ endothelial cells, 5000 Lectin PNA$^-$ EpCAM$^-$ CAFs and 1000 Lectin$^-$ EpCAM$^+$ ductal cells were sorted and further subjected to single-cell capture, barcoding, and reverse transcription using the 10x Genomics platform. (B) UMAP plot of KC and 4KC CAF enriched fractions (9 clusters) and cluster proportions. (C-E) UMAP plot showing previously reported CAF signatures : myCAFs (myofibroblastic CAFs), iCAFs (inflammatory CAFs) and apCAFs (antigen presenting CAFs). Expression scores for each cell were calculated for known CAF subtypes, using previously described signatures (see Methods). The scores were plotted on the same two-dimensional UMAP representation shown in (B). (F-H) Ligand:receptor interaction network was obtained with SingleCellSignalR. (F) chord plots illustrating the number of all paracrine signals between the major cell subtypes (i.e. ducts, CAFs, and PeSCs). (G) heatmaps showing the most variable receptor:ligand interactions among all clusters in KC (left) and 4KC (right) cells. Direction of all pairwise interactions are used to label the columns at the bottom of each heatmap. (H) Detailed Ligand:receptor interactions in KC and 4KC.

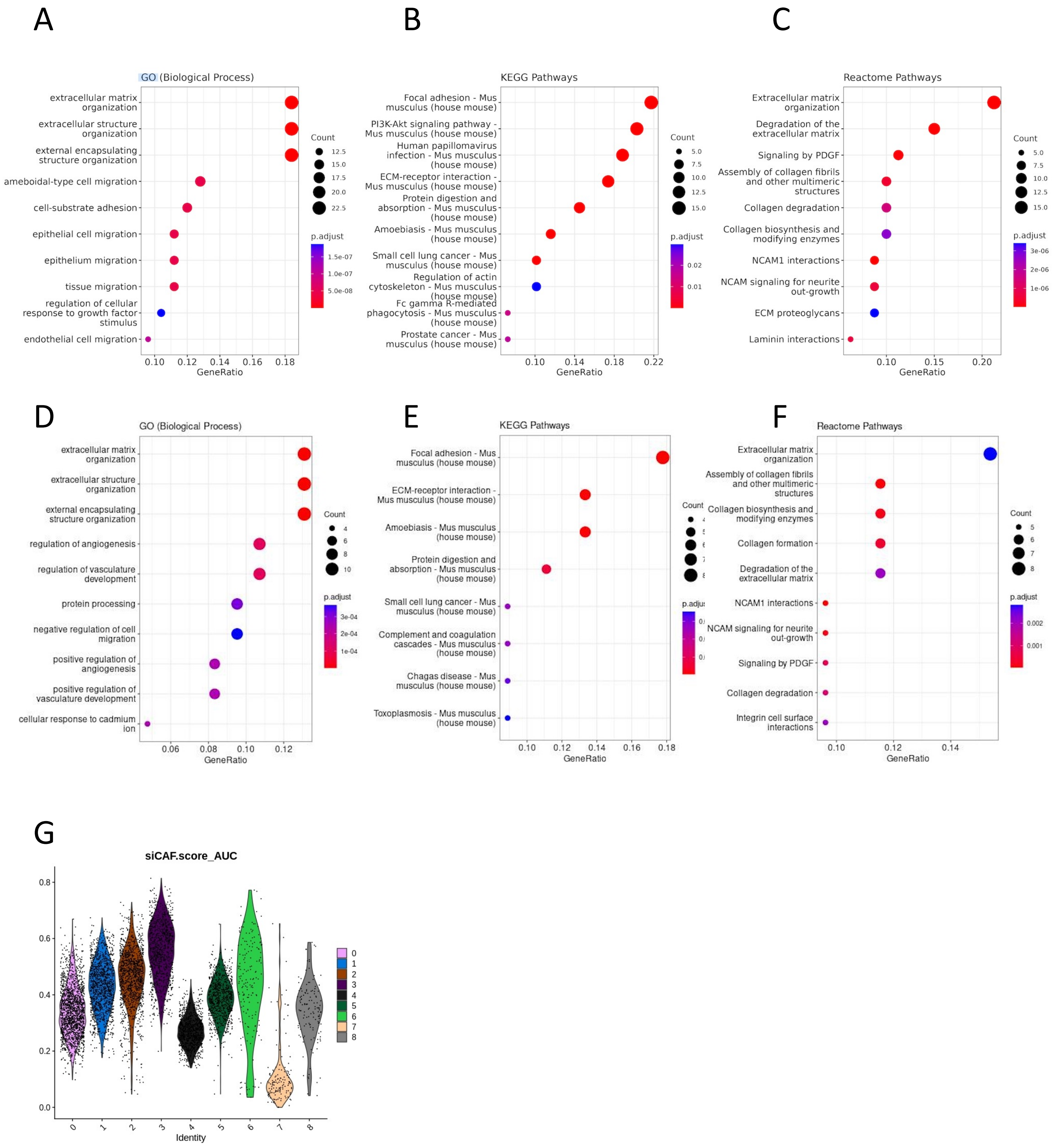

Supplementary Fig. 3

Supplementary Figure 3: Pathway enrichment analysis on single cell data.

(A, B, C). Expression was compared between 4KC and KC condition populations in scRNAseq data subsetted on PDGFRA$^+$ CAFs. (D, E, F). Expression was compared between PDGFRA$^+$ and PDGFRA$^-$ in scRNAseq data subsetted on CAFs. Differentially expressed genes were interrogated for pathway enrichment using different databases (Gene Ontology Biological Process, KEGG and Reactome). The top 10 most significant terms are represented in dotplot charts, where dot color indicates p value and dot size the number of significant genes within each term.

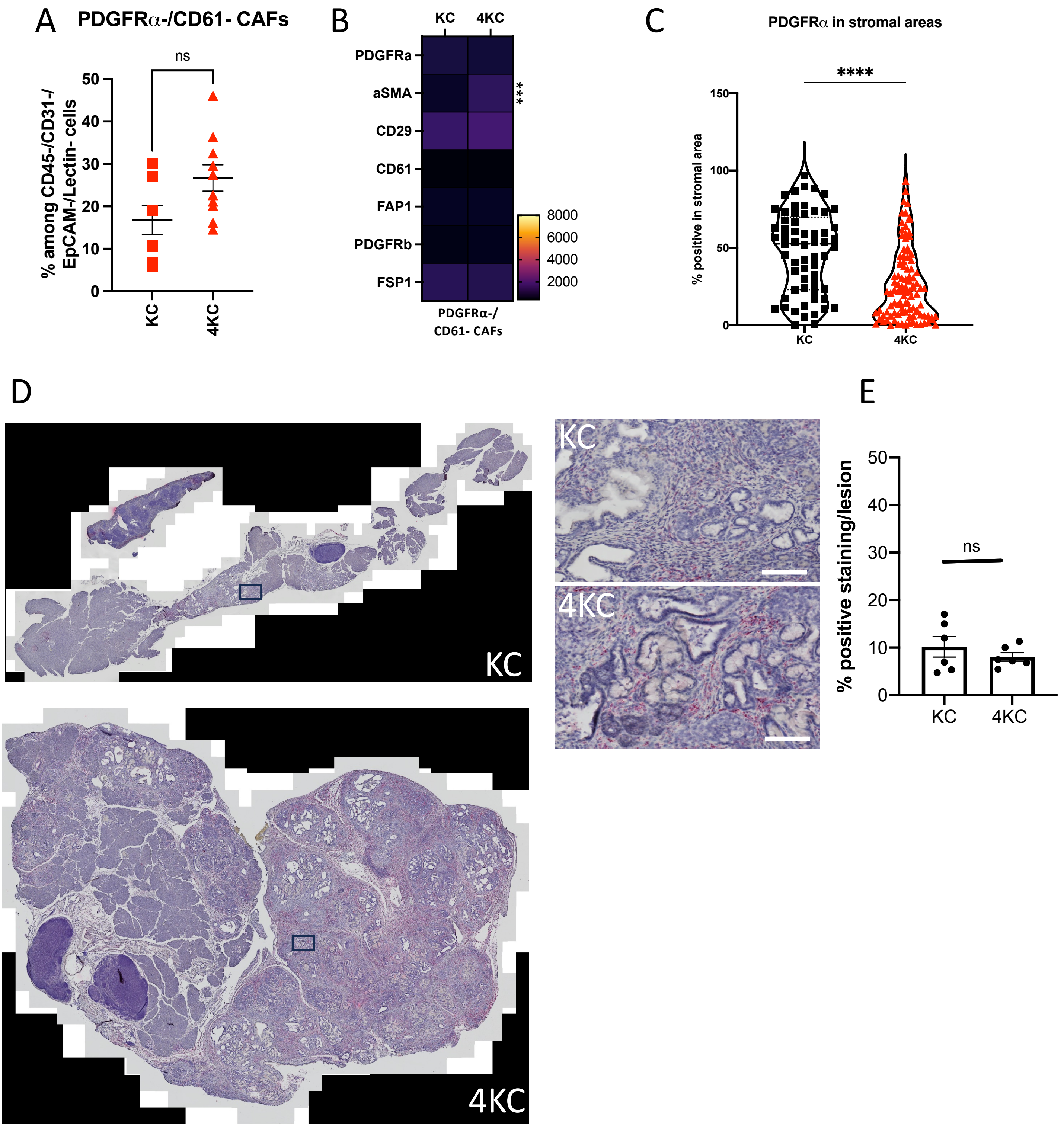

Supplementary Fig. 4

Supplementary Figure 4: Expression of CAF markers on PDFRα^{-}CD61^{-} CAFs.

(A) Frequencies of PDGFRα^{-}CD61^{-} CAFs among CD45^{-}CD31^{-}Lectin PNA^{-}EpCAM^{-} CAFs in pancreata from six-week-old KC (squares) and 4KC mice (triangles). (B) Heatmap of the mean fluorescence intensities (MFIs) of CAF markers on PDGFRα-CD61- CAFs from KC and 4KC pancreata. (C) Quantification of the PDGFRα staining in stromal area in six-week-old KC and 4KC mice. Cumulative data from three individual experiments with three to four mice per group are shown. The mean values SEMs are displayed. ****p <0.0001. (D) Representative scans of PDGRFα RNAscope in sections of pancreata from six-week-old KC and 4KC mice (E) Quantification of the mRNA signal expressed as % per lesion (3 individual mice were used in each group).

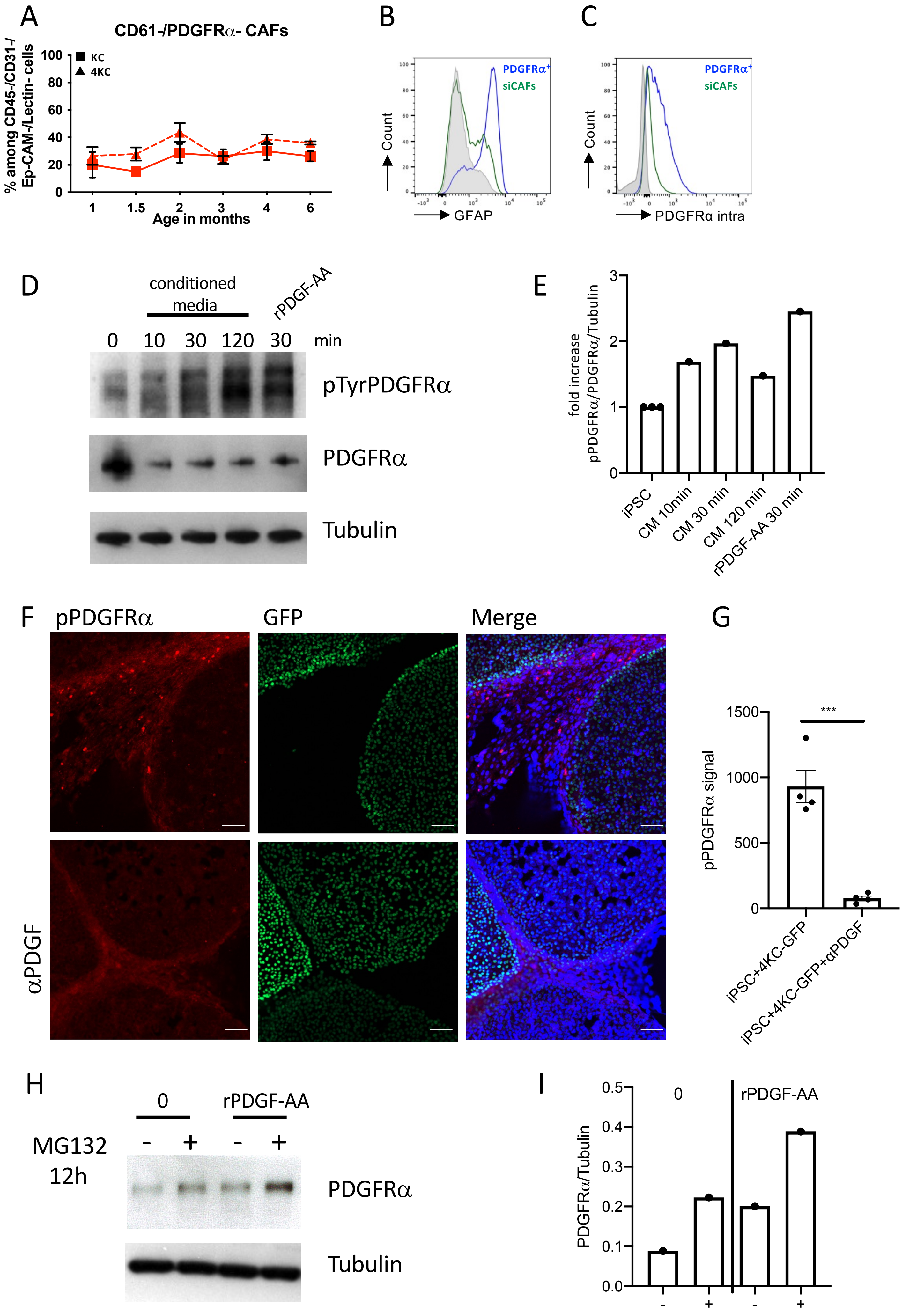

Supplementary Fig. 5

Supplementary Figure 5: ALK4 signaling disruption in tumor cells drives the loss of PDGFR and GFAP expression in siCAFs.

(A) Frequencies of PDGFRα$^-$CD61$^-$ CAFs among CD45$^-$CD31$^-$Lectin PNA$^-$EpCAM$^-$ CAFs determined by FACS analysis of pancreata harvested from KC (squares) and 4KC mice (triangles) at 1, 1.5, 3, 4, and 6 months of age. (B,C) FACS analysis of intracellular GFAP (B) and PDGFRα expression (C) in PDGFRα$^+$ CAFs (blue line) and siCAFs (green line). FMO controls are shown in gray. (A) Results from five mice per group and timepoint are shown. (B, C) Representative data from two individual experiments with five mice are shown. The mean values □ SEMs are displayed. (D) iPSC were cultured for the indicated time points and in presence of 4KC-conditioned media or recombinant PDGF-AA and subjected to WB analysis for pPDGFRα, PDGFRα and tubulin expression. (E) Quantification of the WB signal band intensity presented in (D). (F) iPSC were cultured in the presence or absence of MG-132 for 12h (G) Quantification of the WB signal band intensity presented (F). Representative data from two individual experiments. (H) Representative photographs of PDGFRα phosphorylation and (I) signal quantification after 7 days of iPSC and 4KC-GFP cell line coculture.

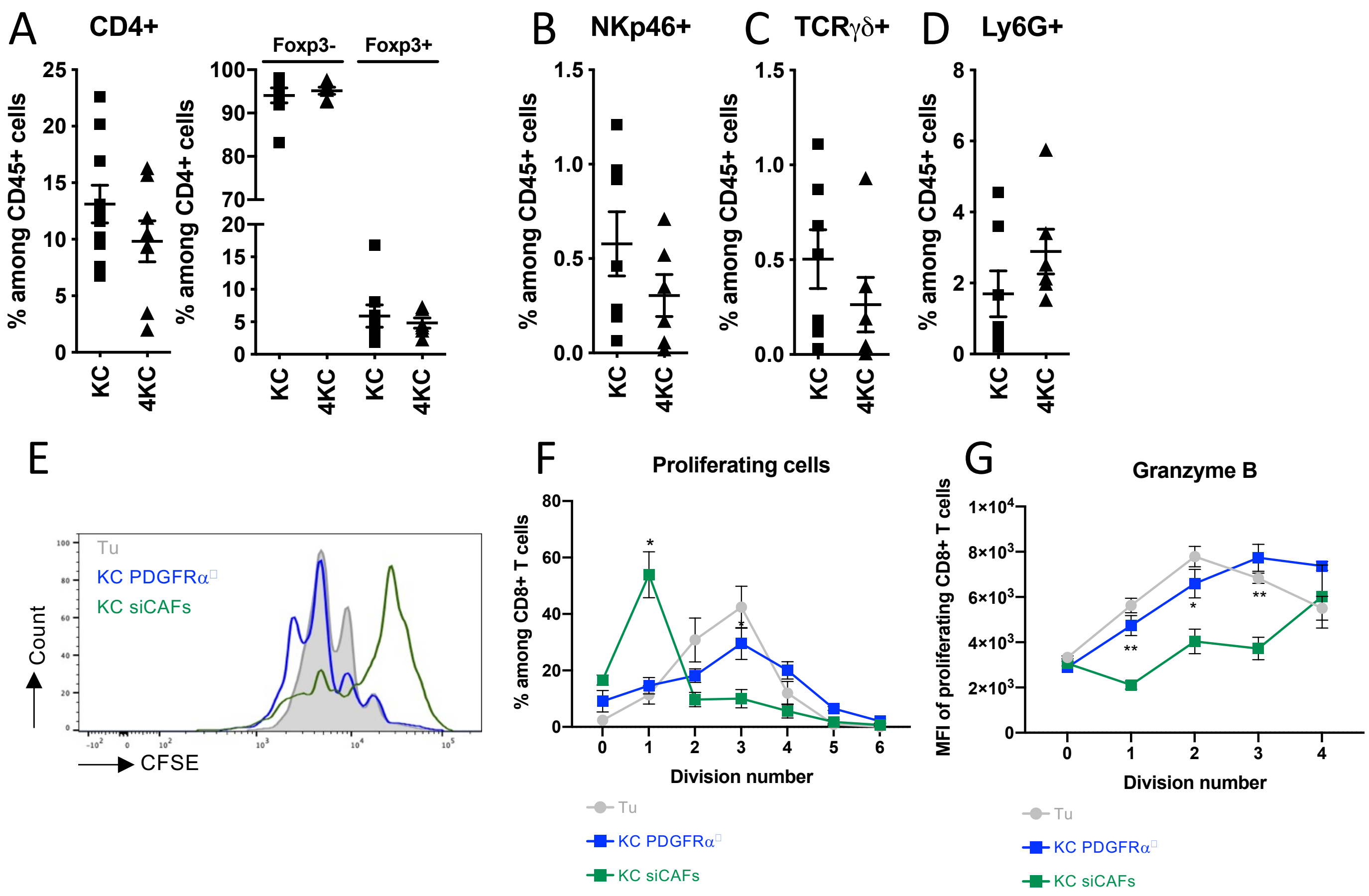

Supplementary Fig. 6

Supplementary Figure 6: CD8$^+$ T-cell activation is impaired by siCAFs. (A) Frequencies of CD4$^+$ T cells among CD45$^+$ cells and Foxp3$^-$ and Foxp3$^+$ cells among CD45$^+$CD4$^+$ T cells in pancreata from 6-week-old KC and 4KC mice. (B-D) Frequencies of NKp46$^+$ (B), TCR$\gamma\delta^+$ (C), and Ly6G$^+$ (D) cells among CD45+ cells in pancreata from KC and 4KC mice. Cumulative data from at least two individual experiments with 3-4 mice per group are shown. (E) CFSE dilution in CD8$^+$ T cells cocultured with BMDCs and CD3/CD28 activation beads in PDGFRα$^+$ CAF/tumor cell-(blue line) or siCAF/tumor cell-conditioned medium (green line). The FMO control is shown in gray. (F, G) Proliferating (F) and Granzyme B (G)-producing CD8$^+$ T cells at the indicated division numbers after coculture with BMDCs and CD3/CD28 activation beads in PDGFRα$^+$ CAF/tumor cell- (blue line) or siCAF/tumor cell-conditioned medium (green line). The FMO control is shown in gray. Representative data from two individual experiments with technical replicates are shown. PDGFRα$^+$ CAFs and siCAFs were isolated from three six-week-old 4KC mice. *$p < 0.05$; **$p < 0.01$.

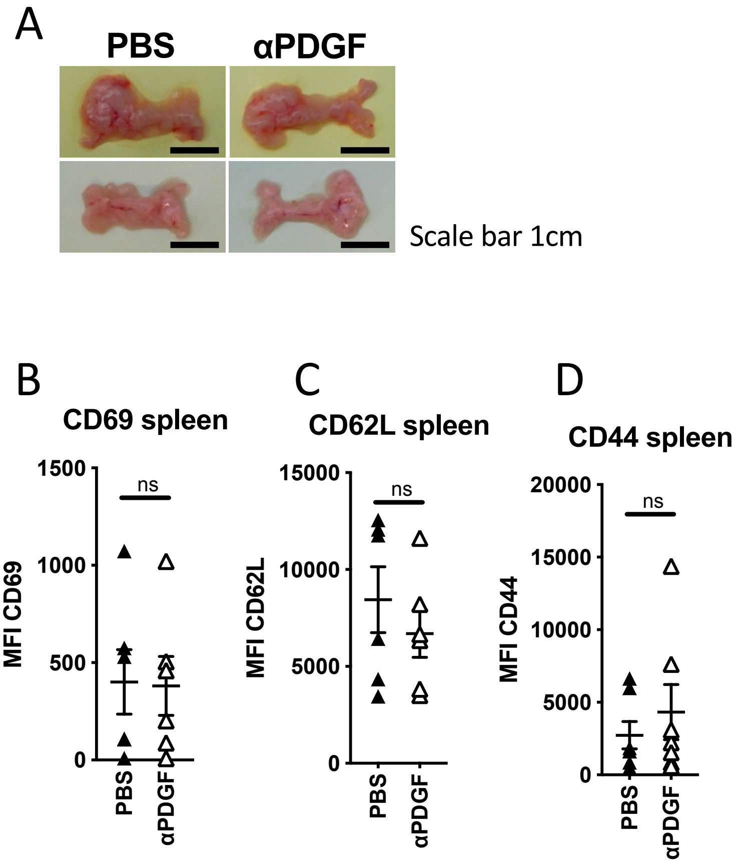

Supplementary Fig. 7

Supplementary Figure 7: PDGF neutralization reduces tumor growth.

(A) Representative photographs of pancreata from six-week-old 4KC littermates treated with an anti-PDGF antibody (right panel) or PBS (left panel). (b-d) FACS analysis of MFIs of CD69, (B), CD44 (C) and CD62L (D) and on $CD8^+$ T cells from the pancreas of six-week-old 4KC mice treated with the anti-PDGF antibody or PBS. ns- not significant.
